# Targeted Metabolic Profiling of Methionine Cycle Metabolites and Redox Thiol Pools in Mammalian Plasma, Cells and Urine

**DOI:** 10.3390/metabo9100235

**Published:** 2019-10-18

**Authors:** Sidney Behringer, Victoria Wingert, Victor Oria, Anke Schumann, Sarah Grünert, Artur Cieslar-Pobuda, Stefan Kölker, Ann-Kathrin Lederer, Donald W. Jacobsen, Judith Staerk, Oliver Schilling, Ute Spiekerkoetter, Luciana Hannibal

**Affiliations:** 1 Laboratory of Clinical Biochemistry and Metabolism, Department of General Pediatrics, Adolescent Medicine and Neonatology, Faculty of Medicine, Medical Center, University of Freiburg, 79106 Freiburg, Germany; sidney.behringer@uniklinik-freiburg.de (S.B.); victoria.wingert@uniklinik-freiburg.de (V.W.); anke.schumann@uniklinik-freiburg.de (A.S.); 2 Institute of Surgical Pathology, Faculty of Medicine, Medical Center, University of Freiburg, 79106 Freiburg, Germany; voria87@gmail.com (V.O.); oliver.schilling@mol-med.uni-freiburg.de (O.S.); 3 Spemann Graduate School of Biology and Medicine, Faculty of Biology, University of Freiburg, 79104 Freiburg, Germany; 4 Department of General Pediatrics, Adolescent Medicine and Neonatology, Faculty of Medicine, Medical Center, University of Freiburg, 79106 Freiburg, Germany; sarah.gruenert@uniklinik-freiburg.de (S.G.); ute.spiekerkoetter@uniklinik-freiburg.de (U.S.); 5 Nordic European Molecular Laboratory (EMBL) Partnership, Centre for Molecular Medicine Norway, University of Oslo, 0318 Oslo, Norway; artur.cieslar-pobuda@ncmm.uio.no (A.C.-P.); judith.staerk@ncmm.uio.no (J.S.); 6 Center for Pediatrics and Adolescent Medicine, Division of Pediatric Neurology and Metabolic Medicine, University Hospital Heidelberg, 69120 Heidelberg, Germany; Stefan.Koelker@med.uni-heidelberg.de; 7 Center for Complementary Medicine, Institute for Infection Prevention and Hospital Epidemiology, Faculty of Medicine, Medical Center, University of Freiburg, 79106 Freiburg, Germany; ann-kathrin.lederer@uniklinik-freiburg.de; 8 Department of Cardiovascular and Metabolic Sciences, Lerner Research Institute, Cleveland Clinic, Cleveland, OH 44106, USA; jacobsd@ccf.org; 9 Norwegian Center for Stem Cell Research, Department of Immunology, Oslo University Hospital, 0372 Oslo, Norway

**Keywords:** methionine metabolism, homocysteine, glutathione, targeted metabolic profiling, mass spectrometry, thiol, biomarker

## Abstract

The concentration of thiol and thioether metabolites in plasma has diagnostic value in genetic diseases of B-vitamin metabolism linked to methionine utilization. Among these, cysteine/cystine (Cys/CSSC) and glutathione/oxidized glutathione (GSH/GSSG) act as cellular redox buffers. A new LC-MS/MS method was developed for the simultaneous detection of cystathionine (Cysta), methionine (Met), methionine sulfoxide (MSO), creatinine and the reduced and oxidized pairs of homocysteine (Hcy/HSSH), cysteine (Cys/CSSC) and glutathione (GSH/GSSG). A one-step thiol-blocking protocol with minimal sample preparation was established to determine redox thiol pairs in plasma and cells. The concentrations of diagnostic biomarkers Hcy, Met, Cysta, and Cys in a cohort of healthy adults (*n* = 53) agreed with reference ranges and published values. Metabolite concentrations were also validated in commercial samples of human, mouse, rat and Beagle dog plasma and by the use of a standardized ERNDIM quality control. Analysis of fibroblasts, endothelial and epithelial cells, human embryonic stem cells, and cancer cell lines showed cell specificity for both the speciation and concentration of thiol and thioether metabolites. This LC-MS/MS platform permits the fast and simultaneous quantification of 10 thiol and thioether metabolites and creatinine using 40 µL plasma, urine or culture medium, or 500,000 cells. The sample preparation protocols are directly transferable to automated metabolomic platforms.

## 1. Introduction

Thiol and thioether metabolites are a group of low-molecular weight molecules involved in one-carbon metabolism, amino acid biosynthesis, redox homeostasis, and transsulfuration reactions. Thiols and thioethers connect central pathways of metabolism by serving as substrates for enzymatic reactions (methionine, homocysteine, cysteine, cystathionine) [1], redox buffers (cysteine, glutathione) [2,3] and in the post-translational modification of cysteine residues in proteins (S-homocysteinylation, S-glutathionylation, S-cysteinylation) [4,5,6]. The concentration of thiols and their disulfides in biological systems is controlled by compartmentalization and by dedicated enzymes that ensure the maintenance of appropriate concentrations and redox state (oxidized and reduced) [7]. Mutations in genes encoding enzymes of the methionine, cobalamin, folate and transsulfuration pathways lead to elevated or reduced concentrations of enzymatically-produced thiol and thioether metabolites, which have diagnostic value. Nutritional deficiency of vitamins B6 (pyridoxine), B9 (folic acid) and B12 (cobalamin), which serve as cofactors in the above-mentioned pathways, also manifest in elevated or reduced concentrations of these metabolites [8,9,10,11]. Inherited diseases of metabolism that are characterized by oxidative stress feature abnormal concentrations of thiol and thioether metabolites, such as glutathione and cysteine [12]. Apart from nutritional deficiencies and inherited disorders of metabolism, metabolites such as methionine and glutathione modulate T cell activation and metabolic reprogramming [13,14,15,16]. In pathology, the concentration of certain thiol and thioether metabolites has predictive value to assess mortality and morbidity in chronic disorders, such as cardiovascular disease [17,18] and cancer [19,20].

Due to their reactivity, thiol and thioether metabolites can be found in several states under physiological conditions, namely, the free thiol pool including reduced and oxidized metabolites, the free mixed disulfide pool and the protein-bound disulfide pool. Methods for the assessment of these three major pools continue to be developed and often require strict sample pre-analytics and thiol derivatization. Detection of thiol and thioether metabolites has exploited liquid chromatography with fluorescence detection, liquid or gas chromatography coupled to mass spectrometry and capillary electrophoresis [21,22,23,24,25,26,27,28,29,30,31,32,33,34]. One major experimental challenge is to prevent thiol oxidation during sample preparation and the enzymatic degradation of the major low molecular weight antioxidant, glutathione. An excellent contribution to solve this problem was made by Jones and colleagues over two decades ago for the accurate quantification of oxidized and reduced glutathione in plasma [35]. Sample preparation consisted of the use of borate buffer (pH 8.5) supplemented with the amino acid serine, to form a borate•Ser complex that inhibits the enzymatic degradation of glutathione by γ-glutamyltranspeptidase [35,36]. This strategy was combined with the inclusion of a thiol blocking agent, and the subsequent derivatization to generate fluorescent thiol-conjugates that were detected by HPLC with fluorescence detection [35,36]. Herein, we adopted and modified the sample pre-analytics procedure of Jones et al. [35] and developed a fast method for the simultaneous quantification of a mixture of thiol and thioether metabolites with diagnostic and prognostic value. We describe a LC-MS/MS method for the simultaneous quantification of cysteine (Cys), cystine (CSSC), homocysteine (Hcy), homocystine (HSSH), reduced glutathione (GSH), oxidized glutathione (GSSG), cystathionine (Cysta), methionine (Met), and methionine sulfoxide (MSO). The non-sulfur biomarker creatinine (Crea) has recently been shown to add power to the assessment of B-vitamin status when used in combination with Hcy and Cys in human serum [18,19]. The steady state concentrations of metabolites Hcy, Cys and Crea result from the partition of Hcy via the B9- and B12-dependent Met cycle to produce SAM as well as the B6-dependent transsulfuration reactions that produce Cys. Formation of Crea depends on the cellular status of SAM, and so does the enzymatic activity of B6-dependent cystathionine β-synthase where SAM serves as an allosteric modulator.

We hypothesized that thiol and thioether metabolites could be simultaneously separated and detected by LC-MS/MS in their oxidized, reduced and thioether forms. Furthermore, we hypothesized that a one-step thiol group derivatization would enable the simultaneous detection of these thiol and thioether metabolites. Our targeted metabolomic method requires a sample preparation time of 25 min, a running time of 5 min and it is suitable for the determination of thiol and thioether metabolites and creatinine in plasma, cells, conditioned cell culture medium and urine. To our knowledge, this is the first fully quantitative LC-MS/MS method to simultaneously determine all key metabolites of the methionine cycle, transsulfuration pathway and creatinine with the inclusion of oxidized and reduced thiol pools, with reduced use of a biological specimen, and a sample preparation protocol directly transferable to automated metabolomic platforms.

## 2. Materials

### 2.1. Materials

Water and all organic solvents were of HPLC and mass spectrometry grade (Honeywell, Germany). Homocysteine (Hcy), homocystine (HSSH), cysteine (Cys), cystine (CSSC), methionine (Met), methionine sulfoxide (MSO), cystathionine (Cysta), glutathione (reduced: GSH, and oxidized: GSSG), iodoacetamide (IA), dithiothreitol (DTT), *S*-methylglutathione (GSMe) and creatinine were purchased from Sigma (Merck KGaA; Darmstadt, Germany) and used without further purification. Identical isotopically labeled standards used to test metabolite quantification versus GSMe were obtained from CDN isotopes (D4-Hcy, D4-Cysta, D4-Met,^13^C_3_-Cys) and Sigma (^15^N-^13^C_2_-GSH and D3-Crea). The analytical performance of the biomarker Hcy in the serum and plasma samples was assessed with external quality control, the Control Special Assays in Serum, European Research Network for the evaluation and improvement of screening, Diagnosis and treatment of Inherited disorders of Metabolism (ERNDIM) IQCS, SAS-02.1 and SAS-02.2 from MCA Laboratories, Winterswijk, Netherlands). Commercially available plasma samples were obtained from Innovative Research, Inc (Michigan, USA) and included Beagle plasma K2-EDTA (product Nr. IGBG-K2EDTA-25918), pooled normal human plasma K2-EDTA (product Nr. IPLA-K2EDTA-23928), Sprague Dawley Rat Plasma K2-EDTA (product Nr. IGRT-K2EDTA-25746), CD1 mouse plasma K2-EDTA (product Nr. IGMS-K2EDTA-25863). Protease inhibitor cocktail for mammalian cell lysates was purchased from Sigma (product Nr. P8340-5ML). Dulbecco’s phosphate-buffered saline (DPBS) was purchased from Thermo Fischer Scientific (product Nr. 14040141, Waltham, MA, USA).

### 2.2. Lysis Buffer

Lysis buffer was prepared by supplementing DPBS with 1% protease inhibitor cocktail on the day of the experiment. Cells were disrupted by freeze-thawing and vortexing. No detergents were used.

### 2.3. Aminothiol Preserving Solution (APS)

An aminothiol preserving solution was prepared with borate buffer (0.1 M, pH 8.5), 0.1 M serine, 10 mM iodoacetamide (thiol blocking agent), and 50 µM GSMe (as internal standard). Aliquots of 0.5 mL APS were stored at −80 °C and were stable for up to 1 year. The composition of APS was modified from Jones et al. [35], with the inclusion of iodoacetamide as a thiol-blocking agent and GSMe as an internal standard (IS). Reaction of thiolate groups with iodoacetamide results in the alkylation of the sulfur-atom to their respective carbamidomethyl modified forms adding a mass of + 57.07 Da.

## 3. Methods

### 3.1. Cell Culture

Human skin fibroblasts, namely, neonatal human dermal fibroblasts (NHDF, Lonza, Basel, Switzerland), human foreskin fibroblasts (HFF, Lerner Research Institute, Cleveland Clinic, Cleveland, OH, USA, described in [37]), GM13395 (skin fibroblast carrying mutations in the methylentetraydrofolate reductase gene, *mthfr*, Coriell Institute for Medical Research, Camden, NJ, USA) were grown in Advanced DMEM (Gibco) supplemented with 10% FBS, 2 mM Glutamax, and antibiotics (100 units/mL penicillin and 100 µg/mL streptomycin). Bovine aortic endothelial cells (BAEC, ATCC, Manassas, VA, USA) and liver hepatocellular carcinoma cells (HepG2, ATCC, Manassas, VA, USA) were grown in DMEM supplemented with 10% fetal bovine serum (FBS), 2 mM Glutamax and antibiotics in the same concentrations given above. Pancreatic cancer cell lines AsPC-1, MiaPaCa-2 and Panc 05.04 were obtained from the American Type Culture Collection (ATCC). AsPC-1 cells were cultured in RPMI 1640 medium containing 10% fetal calf serum (FCS) and antibiotics as described above. MiaPaCa-2 cells were cultured using Dulbecco’s modified Eagle’s medium (DMEM) supplemented with 10% FCS and antibiotics as described above. Panc 05.04 cells were grown in RPMI medium containing 15% fetal calf serum supplemented with 0.1% insulin and antibiotics as described above. Primary human renal tubular epithelial cells (hRTECs) were isolated from the urine of healthy controls and were immortalized according to a published procedure [38]. Immortalized hRTECs were cultured in DMEM Glutamax (Gibco) supplemented with 10% FBS and antibiotics in the concentrations detailed above. Cell culture and immortalization protocols were performed after obtaining informed consent and in compliance with the Declaration of Helsinki. The female human embryonic stem cell (hESC) line WA#22 was obtained from the WiCell Research Institute (Madison, WI, USA) and cultured on mitomycin-C treated mouse embryonic fibroblasts (MEFs). hESCs were maintained in DMEM/F-12 (Sigma) media containing 15% fetal bovine serum (Biological Industries), 5% KnockOut Serum Replacement (Gibco), 1% MEM Non-essential Amino Acids (Gibco), 1% GlutaMAX (Gibco), 1% antibiotic-antimycotic solution (Gibco), 55 μM 2-mercaptoethanol (Life Technologies), and 5 ng/mL of recombinant human bFGF (Miltenyi Biotec). Cells were passaged using 1 mg/mL collagenase type IV (Life Technologies) solution. Pluripotent stem cells were shipped to Germany as PBS-washed, frozen-thawed (dead) cell pellets. All cells were cultured at 37 °C in a humidified atmosphere containing 5% CO_2_.

### 3.2. Isolation of Red Blood Cells (RBCs)

Blood drawn from a healthy adult volunteer was divided into three experimental batches, each held in an unopened EDTA-collection tube. The first batch was processed immediately after blood draw (samples labeled as fresh), and the second and third batches were maintained at room temperature (samples labeled as RT) and 4 °C (samples labeled 4 °C) for 24 h prior to sample preparation. This experiment was designed to mimic common laboratory to laboratory transportation delays to investigate whether this could impact the profile and relative abundance of thiol and thioether metabolites. Red blood cells were isolated by centrifugation at 4200 rpm for 5 min. Cells were washed with PBS and subjected to sample preparation as is described below.

### 3.3. Sample Handling and Storage

#### 3.3.1. Untreated Plasma

Plasma samples for total aminothiol determination were obtained by centrifugation of 1 mL EDTA-blood at 9447× *g* for 1–3 min at room temperature. Plasma was pipetted into a clean 1.5 mL safe-lock Eppendorf tube and stored at −80 °C. Serum samples were handled and stored in the exact same way after spontaneous separation of serum from cells. Plasma samples chosen for reduced and oxidized aminothiol determination were handled as described under the Sample Pre-analytics and Preparation section.

#### 3.3.2. APS-Treated Plasma

Plasma samples destined for oxidized and reduced thiol determination were collected from freshly drawn blood treated with an equal volume of APS, mixed three times by inversion, and centrifuged at 9447× *g* for 5 min at room temperature. These plasma samples are hereafter referred to as APS-plasma. APS-plasma samples were frozen in dry-ice and stored at −80 °C. Carbamidomethyl-derivatives of Hcy, Cys and GSH in APS-plasma stored at −80 °C were stable for up to 18 months.

#### 3.3.3. Urine

Fresh, (maximum 3 h post-collection) spontaneous urine was received and stored at −80 °C without modification. When the samples required lab to lab transportation, this was done in dry-ice at all times. For the determination of aminothiols, urine was used without dilution. For the determination of creatinine, urine was diluted 1:50 with water. The analytical performance of creatinine detection was cross-validated in a subset of urine samples collected from healthy individuals. Creatinine in freshly collected urine was analyzed externally at the Central Diagnostic Laboratory of the University Hospital Freiburg using an automated kinetic Jaffe method, in parallel with our LC-MS/MS method, and the concentrations obtained by these independent measurements were compared.

#### 3.3.4. Cells

Cells isolated for total as well as the reduced and oxidized aminothiol determinations were harvested by trypsinization, washed 1x with DPBS and stored as dry-cell pellets at −80 °C. Lab to lab transportation of frozen cell specimens was performed on dry-ice.

#### 3.3.5. Conditioned Culture Medium

Conditioned (spent) culture medium was collected and the cells and debris were removed by centrifugation at 9447× *g* for 10 min. Cleared conditioned culture medium was transferred to a clean 1.5 mL safe-lock Eppendorf tube and stored at −80 °C for further use.

### 3.4. Calibration Curves

#### 3.4.1. Total Reduced Thiols

Calibration curves consisted of 12 points (calibrators 1–12) selected to cover the range of expected physiological concentrations of each metabolite in biological specimens. A master mix was prepared freshly on the day of the experiment from individual stocks of each metabolite (1 mM each, prepared in water and stored at −80 °C for up to 12 months). Serial dilutions were made from that master mix, to a total of 12 dilutions. Calibration curves were made as follows: Hcy 0–100 µM, Cysta and HSSH 0–50 µM, GSH 0–20 µM for plasma and GSH 0–500 µM for cells, GSSG 0–10 µM, Cys 0–300 µM, and CSSC, Met, MSO 0–150 µM and 0–500 µM Crea. For total aminothiol determination sample preparation was made up of 20 µL calibrators 1-12 that was mixed with 20 µL IS and 20 µL H_2_O.

#### 3.4.2. Oxidized, Reduced and Total Thiol Pools

In experiments where free reduced and oxidized aminothiols were determined, a second calibration curve was included identically to the one described above, except that sample preparation was made by mixing 20 µL calibrator, 20 µL APS solution (containing IS) and 20 µL H_2_O. This second calibration curve converts GSH, Hcy and Cys into their carbamidomethylated forms for the quantification of free-reduced thiols in biological samples.

### 3.5. Internal Standard Solution

The internal standard consisted of 50 µM GSMe, 50 µM each of D4-Hcy, D4-Cysta, D4-Met, ^13^C_3_-Cys and ^15^N-^13^C_2_-GSH, and 10 µM D3-Crea prepared in water. GSMe is structurally compatible with the thiol and thioether species to be detected, unreactive toward components of plasma, cells and urine and stable under the conditions of sample preparation.

### 3.6. LC-MS/MS Method

Metabolites were separated on a SunFire C8 Reversed-Phase column with 3.5 µm spherical silica (particle size 100 Å), 4.6 mm × 150 mm (Waters, Milford, MA, USA) using an isocratic method of 95% solvent A (0.1% formic acid in water) and 5% B (0.1% formic acid in MeOH) with a flow of 0.75 mL/min over a period of 5 min. Metabolites were detected on a Sciex 6500+ ESI-tripleQ MS/MS on low mass mode (0–1000 Da), with curtain gas (CUR) at 40, collision gas (CAD) at 10, Ion spray voltage (IS) at 5000 Volts, temperature (TEM) at 400 °C, ion source gas 1 (GS1) at 40 and ion source gas 2 (GS2) at 30. Each metabolite was optimized individually using chromatographic solvent conditions. All metabolites exhibited optimal ionization in the positive mode.

#### 3.6.1. Analysis of Matrix Effects

The effect of matrix was assessed by comparing the peak areas of the respective metabolites in the calibration curves made up in water versus those where the solvent was the extracted matrix, i.e., plasma, cell lysate or urine, according to published procedures [39,40,41]. Both isotopically labeled metabolites and GSMe were used as internal standards. Variation in the determination of Hcy concentration in a commercial quality control sample containing a known concentration of Hcy (Control Special Assays in Serum, ERNDIM, SAS-02.2 from MCA Laboratories, Winterswijk, Netherlands) as well as in commercially available pooled human plasma from healthy individuals (Innovative Research, Inc, Michigan, USA product Nr. IPLA-K2EDTA-23928) was evaluated using the calibration curves prepared with the different matrices. Homocysteine was chosen due to its known susceptibility to matrix effects [42] and also because it is a widely used biomarker for diseases of methionine, folate and transsulfuration metabolism. In a second approach, a side-by-side comparison of the performance of metabolite quantification using identical isotopically labeled standards for Hcy, Cys, Cysta, GSH and Met versus the generic internal standard GSMe was carried out.

#### 3.6.2. Stability, Recovery and Carry Over

The stability of metabolites was investigated using two different approaches. Firstly, freshly prepared solutions of commercially available metabolites were subjected to sample preparation immediately or after incubation at room temperature for 60 min. Peak areas of metabolites subjected to sample preparation without delay and after 60 min were compared. A comparison of metabolite concentrations during this time-frame showed less than 5% variation. Secondly, the concentration of metabolites present in freshly isolated human plasma (endogenous) was monitored at 0, 15, 30, and 60 min post-isolation to investigate their stability in the presence of the biological matrix. Recovery was examined by spiking a known amount of isotopically labeled metabolites into the biological sample and comparing the recovered concentrations with respect to those of a sample of water spiked under identical conditions. Carry over under our chromatographic conditions was examined by a blank injection (water) right after injecting the calibrator sample with the highest metabolite concentrations. Blank injections were also included at the end of a sequence of biological sample injections. No carry over was detected under the experimental conditions, regardless of the biological matrix analyzed.

#### 3.6.3. Validation

This methodology followed the guidelines set forth by the European Medicines Agency for the validation of analytical procedures Q2 (R1) [43].

### 3.7. Sample Pre-Analytics and Preparation

Flow-diagrams of sample pre-analytics and preparation for each biological specimen are provided in supplemental data (Figures S1–S3). Detailed descriptions follow below.

#### 3.7.1. Total Thiol and Thioether Metabolites in Untreated Plasma

Untreated plasma is routinely used in diagnostic laboratories to quantify total concentrations of Hcy, Cys, Cysta, Met and Crea, which are then referred to standardized reference ranges in healthy subjects. In this protocol, protein-bound, free oxidized thiols and mixed disulfides are all converted to their free reduced forms. Briefly, 20 µL plasma was mixed with 20 µL IS and 20 µL DTT 0.5 M, vortexed and incubated for 10 min at room temperature. Proteins were precipitated by the addition of 100 µL of 0.1% formic acid in MeOH, followed by centrifugation at 9447× *g* for 10 min at room temperature. Supernatants were transferred to HPLC vials for LC-MS/MS analysis or stored at −80 °C for further analysis (samples were stable for at least 1 month).

#### 3.7.2. Stability of Thiol and Thioether Metabolites in Freshly isolated Untreated Plasma

Plasma was isolated from freshly-drawn blood from 4 healthy subjects as described above. Four separate aliquots of plasma were set aside for each individual. The first set was subjected to sample preparation without delay (time = 0 min). The other 3 plasma aliquots were processed at 15, 30, and 60 min post-blood collection. In order to monitor both endogenous free reduced and free oxidized thiols, the sample preparation protocol did not include DTT. Samples were prepared by mixing 20 µL of plasma with 20 µL internal standard and 20 µL H_2_O. Deproteinization was performed as described above for the quantification of total thiol and thioether metabolites.

#### 3.7.3. Redox Thiol Pools and Thiol and Thioether Metabolites in APS-treated Plasma

Plasma samples were prepared and isolated in the presence of APS as described above. Two aliquots each of 40 µL APS-plasma were incubated separately with either 20 µL H_2_O (this quantifies free reduced thiol pools, i.e., the carbamidomethyl modified forms of GSH, Cys and Hcy, plus free oxidized species GSSG, HSSH and CSSC) or with 20 µL DTT 0.5 M (DTT reduces protein-bound thiols and free oxidized thiols into their free reduced forms), vortexed and incubated for 10 min at room temperature. Proteins were precipitated by addition of 100 µL of 0.1% formic acid in MeOH, followed by centrifugation at 9447× *g* for 10 min at room temperature. Supernatants were transferred to HPLC vials for LC-MS/MS analysis or stored at −80 °C for further analysis (samples were stable for at least 1 month).

#### 3.7.4. Total Thiol and Thioether Metabolites in Untreated Cell Lysates

Cultured cells (0.25–1 × 10^6^ cells) were harvested by trypsinization, washed 2 times with DPBS, and the cell pellets were frozen at −80 °C until further analysis. On the day of sample analysis, cell pellets were thawed and quickly resuspended with 200 µL lysis buffer. A 20 µL aliquot of cell suspension was mixed with 20 µL of IS solution and 20 µL of DTT. The remaining cell lysate was stored at −80 °C for total protein quantification to normalize metabolite concentrations or for additional studies. The sample was then subjected to 3 alternating freeze-thawing cycles in dry ice (5 min on dry-ice, 5 min at room temperature) vortexing between freeze-thawing cycles. Proteins were precipitated by the addition of 100 µL of 0.1% formic acid in MeOH, followed by centrifugation at 9447× *g* for 10 min. Supernatants were transferred to HPLC vials for LC-mass spectrometry analysis or stored at −80 °C for further analysis (samples were stable for at least 2 weeks).

#### 3.7.5. Redox Thiol Pools, Thiol and Thioether Metabolites in APS-treated Cells

To determine the concentration of the redox thiol pools, cells (0.25–1 × 10^6^ cells) were harvested, washed 2 times with PBS, and the cell pellets were frozen at −80 °C until further analysis unless otherwise indicated. On the day of the sample analysis, cell pellets were thawed and quickly (less than 1 min per sample) resuspended with 200 µL lysis buffer. Two separate aliquots each of 20 µL of cell suspension was mixed with 20 µL of APS solution and subjected to 3 alternating freeze-thawing cycles in dry ice (5 min on ice, 5 min at room temperature), vortexing between freeze-thawing cycles. The remaining cell lysate was stored at −80 °C for total protein quantification to normalize metabolite concentrations or for additional studies. After freeze-thawing, one set was treated with 20 µL of water in order to quantify blocked plus free oxidized aminothiols, and the second set was treated with 20 µL of 0.5 M DTT to convert free oxidized thiols and protein-bound thiols into their free reduced forms. Samples were incubated at room temperature for 15 min. Proteins were precipitated by the addition of 100 µL of 0.1% formic acid in MeOH, followed by centrifugation at 9447× *g* for 10 min. Supernatants were transferred to HPLC vials for LC-MS/MS analysis or stored at −80 °C for further analysis (samples were stable for at least 1 month).

#### 3.7.6. Total Thiol and Thioether Metabolites in Conditioned (Spent) Culture Medium

Because mammalian cells normally grow in the presence of oxygen (95% humidity air, 5% CO_2_), only total metabolite concentrations can be determined in the conditioned culture medium. An aliquot (0.1–1 mL, depending on culture dish or flask of choice) was taken at the desired time point, centrifuged at 9447× *g* 10 min at room temperature to remove the dead cells and debris, and the cleared spent medium was stored at −80 °C until further analysis. On the day of sample analysis, 20 µL of conditioned medium was supplemented with 20 µL IS and 20 µL 0.5 M DTT. The sample was incubated at room temperature for 10 min. Proteins were precipitated by addition of 100 µL of 0.1% FA in MeOH, followed by centrifugation at 9447× *g* for 10 min. Supernatants were transferred to HPLC vials for LC-MS/MS analysis or stored at −80 °C for further analysis (samples were stable for at least 1 month).

#### 3.7.7. Total Thiol and Thioether Metabolites in Urine

Analysis of metabolites in urine was performed as described for conditioned culture medium. Briefly, 20 µL of urine was mixed with 20 µL IS and 20 µL 0.5 M DTT. The sample was incubated at room temperature for 10 min. Proteins were precipitated by addition of 100 µL of 0.1% FA in MeOH, followed by centrifugation at 9447× *g* for 10 min. Supernatants were transferred to HPLC vials for LC-MS/MS analysis or stored at −80 °C for further analysis (samples were stable for at least 1 month).

### 3.8. Ethical Approval

Informed consent was received for all subjects in this study under ethical approvals EK-Nr. 218/17 and EK-Nr. 198/18, Freiburg, Germany, and clinical trial Nr. DRKS00011963; German Clinical Trial Registry.

### 3.9. Data Analysis

Data analysis was performed with MATLAB 2019a (The MathWorks, Inc, Natick, MA, USA). A minimum of 4 samples were examined per experimental condition. Unless otherwise indicated, all values are expressed as mean ± standard deviation.

## 4. Results

### 4.1. Separation and Quantification of Thiol and Thioether Metabolites by LC-MS/MS

**Figure 1:**
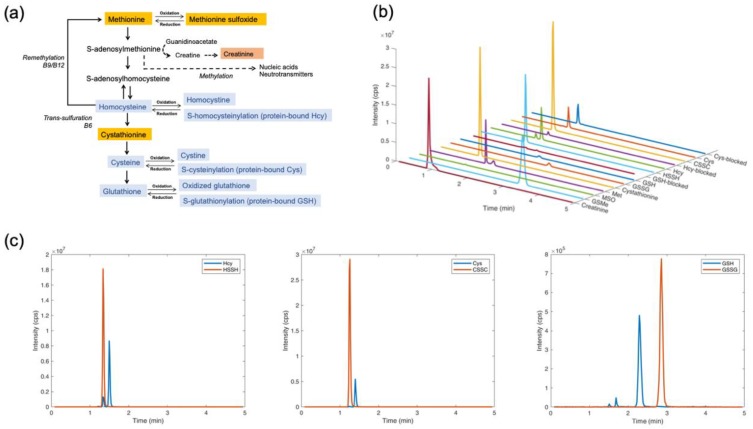
Pathways and chromatograms of the 10 metabolites examined in this study. Panel (**a**). Metabolites and redox species determined in this study are shown in colored boxes. Dietary Met is predominantly converted to *S*-adenosylmethionine (SAM), the universal methyl donor that furnishes nucleic acid and neurotransmitter metabolism. Guanidinoacetate, an intermediate of arginine metabolism, is methylated by SAM to form creatine-phosphate, the precursor of creatinine. Methyl transfer reactions driven by SAM yield *S*-adenosylhomocysteine (SAH), which is in turn converted to homocysteine. Depending on cellular needs, homocysteine can undergo remethylation to Met, conversion back to SAH or enter the transsulfuration pathway for the biosynthesis of cystathionine and cysteine, which represents the first half of the glutathione biosynthesis pathway. Cys, Hcy and GSH exist in free reduced and free oxidized forms, and also bound to proteins in the form of mixed disulfides. Reactions involved in the methionine cycle and transsulfuration routes utilize vitamins B6, B9 (folate) and B12 as cofactors. Deficiencies of these B-vitamins result in characteristic changes in metabolite concentrations, with the common denominator of homocysteine elevation. Panel (**b**). Chromatogram of thiol and thioether metabolites and creatinine. The terms Hcy-blocked, Cys-blocked and GSH-blocked denote the corresponding free-reduced species of homocysteine, cysteine and glutathione upon derivatization with iodoacetamide. Panel (**c**). Chromatogram of oxidized and reduced thiol species of Hcy, Cys and GSH. The metabolites were separated in a total running time of 5 min, using acidic conditions with solvent A 0.1% formic acid in water and solvent B 0.1% formic acid in methanol.

We developed a method for the quantitative assessment of 10 biological thiol and thioether metabolites and creatinine, all of which are involved in B-vitamin metabolism relevant to folate, methionine and transsulfuration pathways (Figure 1a). A chromatogram of all metabolites examined in this study is shown in Figure 1b. Separate chromatograms of oxidized and reduced species of homocysteine, cysteine and glutathione are provided in Figure 1c. All metabolites were detected in the positive mode. Retention times, mass transitions and optimized mass spectrometry parameters for each metabolite are given in Table 1. The metabolites were resolved within 5 min and quantified based on commercially available calibrators and either identical isotopically labeled standards or GSMe as a generic internal standard. Creatinine measurements utilized exclusively D3-creatinine as an internal standard, given that this metabolite differs structurally from the thiol and thioether species quantified herein. With the exception of Met, all metabolites yielded a single, well-defined peak. The doublet peak shape of Met corresponds to its ionic forms, as the pH of the chromatographic conditions (pH 2.2) is in the range of the pKa of the carboxyl moiety of Met (pKa = 2.28 [44]). Integration of the Met double-peak signal yielded a linear correlation, which permitted the accurate determination of its concentration (Table 2 and Figure S4).

### 4.2. Linearity Range and Limit of Quantification

As shown in Table 2, integration of the peak areas resulted in suitable linearity for the quantification of all metabolites in their oxidized, reduced and carbamidomethyl modified forms (+ 57.07 Da). The oxidized thiols exhibited lower limits of quantification compared to the reduced and blocked forms. Calibration curves are given in supporting information Figure S4. Blank injections with water after injecting the calibrator with the highest concentration of metabolites and after running a sequence of biological samples showed no carry over under our chromatographic conditions.

### 4.3. Analysis of Matrix Effects

The effect of matrix on metabolite quantification was evaluated by comparing calibration curves made in water versus calibration curves made with extracted matrices, i.e., plasma, cell lysate and urine, in accord with published recommendations [40]. Calibration curves are provided in Figure S5, Panel (a). As can be observed, calibration curves made with extracted biological matrices do not differ significantly from those made in water under metabolite extraction and chromatographic conditions. A list of slopes from all calibration curves and goodness of fit measures (R^2^) of the linear regressions is provided in Table S2. An exception is the quantification of creatinine in urine, which showed marked dispersion of the data points. This occurs due to the very high endogenous concentration of creatinine in urine. Table S3 shows results of the quantification of Hcy, a metabolite that is susceptible to matrix effects, in a quality control sample (QC, lyophilized serum) and in commercial pooled human plasma using calibrators made in water, or in extracted plasma, cell lysate or urine. The values of Hcy concentration in commercial human plasma with these calibrators were 7.2, 8.5, 8.1, and 7.6 µM, respectively. The calculated concentration of Hcy in the QC was, in all cases, within the expected concentration range provided by the supplier, 44–65 µM.

Next, a side-by-side comparison of the performance of metabolite quantification was carried out using identical isotopically labeled standards for Hcy, Cys, Cysta, GSH and Met versus the generic internal standard GSMe. Under our experimental conditions, the variation in expected concentration values did not exceed 15% between the quantifications with GSMe or an identical stable isotopically labeled standard of Cysta, Met, MSO and GSH. However, up to a 1.7-fold variation was noted for Cys and Hcy. We therefore examined whether the source of variation was matrix effects. Graphs of peak areas of the metabolite divided by peak area of the internal standard in calibrators (prepared in water), quality control for Hcy concentration (QC, lyophilized and reconstituted commercial plasma spiked with a known concentration of Hcy) and human plasma are provided in Figure S5, Panel (b). As can be observed, a significant reduction of the intensity of the internal standard occurs in the presence of QC and plasma. This effect differs between isotopically labeled standards and GSMe, thus explaining the variation between the quantification with identical standards versus GSMe, particularly in the case of Hcy and Cys. Therefore, direct quantification is preferred with identical isotopically labeled internal standards. Alternatively, an adjustment for matrix effect contributions should be made if using GSMe as a generic internal standard. For this, the calibrators and one pooled sample of the matrix under study, for example plasma, should be simultaneously spiked with isotopically labeled standards and GSMe in each experimental run to generate the appropriate adjustment factor that would apply to the entire sample set of that biological matrix. This would reduce sample costs compared to the inclusion of isotopically labeled standards in high-throughput sample runs.

### 4.4. Stability and Recovery of Thiol and Thioether Metabolites in Plasma

**Figure 2:**
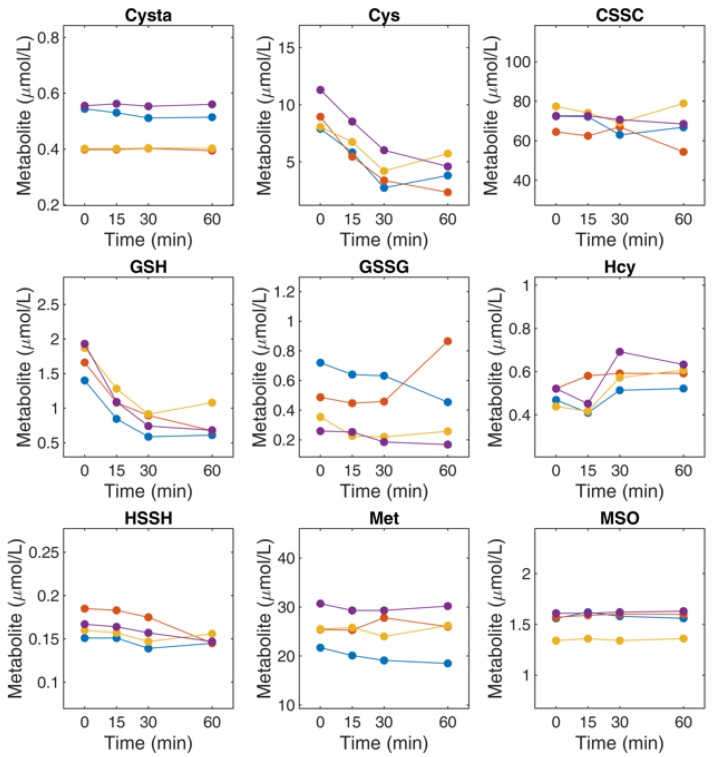
Stability of thiol and thioether metabolites in untreated human plasma. Metabolites were analysed immediately after blood-draw and plasma isolation (t = 0 min) and after 15, 30, and 60 min incubation of blood at room temperature. Cys and GSH underwent marked degradation over the course of this experiment.

All metabolites investigated in this study were stable in the sample preparation solution for at least 6 h at room temperature (less than 0.5% variation in peak areas of calibrators after 6 h of incubation at room temperature). Recovery was examined by spiking a known concentration of commercially available isotopically labeled metabolites into water, plasma, cell lysate and urine and comparing their concentrations after the same sample preparation. Recovery of spiked Crea, Cysta, and Met was 75–95% when comparing plasma, cell lysate and urine versus water (100% recovery). Recovery of spiked Hcy, Cys and GSH was 50–60% in the absence of DTT and > 90% in the presence of DTT. This is consistent with the known property of these metabolites of reacting with cysteine residues in proteins and with other thiols in the biological matrix to form disulfide bonds. Plasma and serum are the most widely used specimens for metabolite-based diagnostics. The concentrations of cysteine and glutathione pools in plasma are sensitive to a number of factors including the time from blood collection to analysis, storage conditions, and the occurrence of hemolysis all of which alter the relative abundance of reduced and oxidized species, and in some cases, lead to the enzymatic degradation of the metabolite of interest. The stability of Met, MSO, Cysta, Hcy, HSSH, Cys, CSSC, GSH and GSSG was examined immediately after plasma isolation and over the course of 60 min at room temperature in 4 healthy subjects. As shown in Figure 2 and Table S1, Cys and GSH exhibited a marked decrease in concentration over 60 min. The remaining metabolites were stable with only very minor changes in concentration observed after 60 min.

### 4.5. Profile of Thiol and Thioether Metabolites as Examined in Diagnostic Platforms

**Figure 3:**
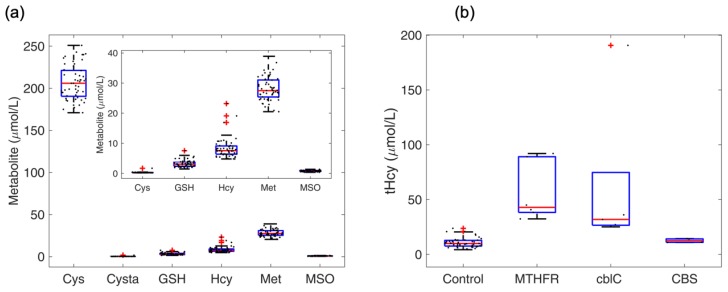
Analysis of thiol and thioether metabolites in untreated human plasma as typically processed in clinical diagnostic laboratories. Panel (**a**). Plasma metabolites in a cohort of healthy individuals (18–59 years old, *N* = 53). Panel (**b**). Plasma homocysteine in healthy subjects and in patients with homocystinuria due to deficiencies in methylenetetrahydrofolate reductase, cblC and cystathionine β-synthase (MTHFR, cblC, CBS). The patient with CBS deficiency was responsive to treatment with vitamin B6, which afforded excellent biochemical control of plasma Hcy concentration to the range observed in healthy subjects (5–15 µM). Center mark (red) represents the median, and bottom and top edges of the box represent the 25th and 75th percentiles, respectively. The whiskers extend to the most extreme data points, and points considered outliers are marked with the ‘+’ symbol. The outliers in the disease groups typically represent patients with a severe phenotype, or else patients with poor compliance with or no response to treatment.

Total concentrations of thiol and thioether metabolites were first examined in untreated plasma, such to compare the performance of our method with reference ranges in accredited clinical chemistry laboratories. Total concentrations of Met, Cys, Hcy, Cysta, GSH and MSO were measured in a cohort of 53 healthy adult subjects (Table S4). As shown in Figure 3a, the values obtained using our LC-MS/MS platform agreed with those reported in a comprehensive literature review [2] as well as by standard laboratory reference ranges in external laboratories (see for example, Bevital, Bergen, Norway, http://www.bevital.no). Next, we subjected the data to correlation analysis for all metabolites. No statistically significant correlations were found among the panel of metabolites examined in this study (Figure S6). Examination of plasma Hcy concentrations in patients diagnosed with genetic homocystinurias, namely, methylentetrahydrofolate reductase (MTHFR) deficiency, and methylmalonic aciduria combined with homocystinuria type C (MMACHC, cblC) resulted in the expected elevations compared to the healthy control group (Figure 3b). Plasma from patients with vitamin B6-responsive cystathionine β-synthase (CBS, B6-responsive, classic homocystinuria) showed near normal values in agreement with the positive response to treatment of this CBS subtype.

### 4.6. Profile of Thiol and Thioether Metabolites in Commercial Human and Animal Plasma

The analysis of thiol and thioether metabolites is also valuable in research. Oftentimes, human diseases modeled in rodents and pharmacological interventions are performed in Beagle dogs prior to clinical trials in humans. We therefore examined the concentration of thiol and thioether metabolites and creatinine in commercial plasma samples from several animal species of relevance to research. As presented in Table 3, substantial differences exist for metabolite concentrations among animal species. Analysis of commercial human plasma yielded results in accord with reported reference ranges. Quality control values for Hcy in human plasma (QC from ERNDIM) were within the expected range of concentration.

### 4.7. Determination of Thiol and Thioether Metabolites in Their Free-reduced, Oxidized and Protein-Bound Forms

**Figure 4:**
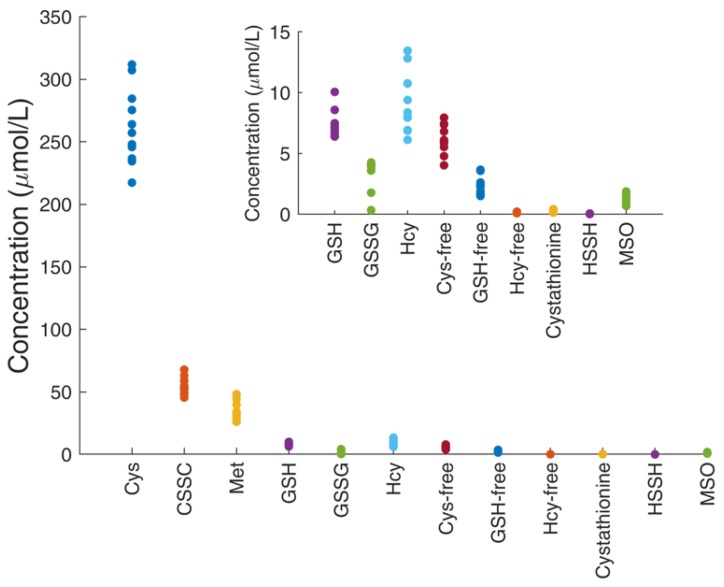
Thiol and thioether metabolites and relevant thiol/disulfide pools in healthy human subjects. Pools of oxidized and reduced cysteine, glutathione and homocysteine and other thiol and thioether metabolites in a cohort of 11 healthy adult subjects were determined in APS-plasma samples. As expected, Cys/CSSC represents the major thiol/disulfide pool in human plasma.

Free reduced and free oxidized species of Hcy, Cys and GSH plasma isolated from freshly-drawn blood treated with APS were determined in a cohort of 11 healthy adult subjects according to sample pre-analytics and preparation described in the Methods section. The analysis also included non-redox labile metabolites Cysta, Met, and MSO. The results are shown in Figure 4 and Table 4. As expected, Cys species make up the most abundant low molecular weight thiol component of human plasma, followed by approximately one order of magnitude lower concentration of GSH and Hcy. The concentrations listed under ‘Cys-free’, ‘Hcy-free’ and ‘GSH-free’ represent the endogenous pools of cysteine, homocysteine and glutathione that exist in the reduced, free form, that is, not involved in disulfide bond formation with other thiols or with cysteine residues of proteins. This pool of free thiols is known to be low as opposed to their total concentrations that include protein-bound pools plus oxidized and free reduced pools (all converted into free reduced thiols by addition of DTT). As detailed in Table 4, the absolute concentrations determined herein for all species and redox pools are in reasonable agreement with those reported in the literature obtained by other methods and research groups [2] (reference ranges are given in the last row of Table 4).

### 4.8. Determination of Total Thiol and Thioether Metabolites and Creatinine in Urine

**Figure 5:**
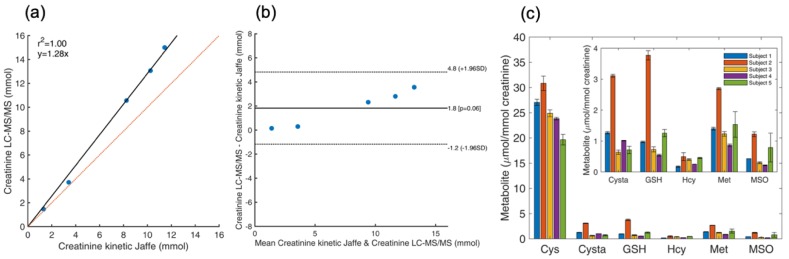
Analysis of thiol and thioether metabolites and creatinine in human urine. Panel (**a**). Correlation of creatinine concentration determined by the kinetic Jaffe method versus our LC-MS/MS platform. The orange line represents the expected fit for perfect correlation of values (slope = 1). Panel (**b**). Brand-Altman plot examining the performance of our LC-MS/MS platform in the determination of urinary creatinine with respect to results on the same samples obtained with the standardized kinetic Jaffe method (Central diagnostic laboratory, Medical Center, University of Freiburg). Differences in discrete concentration values are within 2 standard deviations (±1.96 SD). Panel (**c**). Profile of thiol and thioether metabolites in human urine normalized by creatinine. Values are expressed as the mean ± standard deviation.

Urine is a biological fluid that is more readily available in patients for whom blood draws are not possible or not recommended. All urinary metabolites are typically normalized by the concentration of creatinine, which accounts for renal clearance. Creatinine was examined under identical sample preparation and LC-MS/MS protocols used for thiol and thioether metabolites. The reliability of creatinine determination by our LC-MS/MS method was determined by subjecting a subset of urine samples from 5 healthy subjects to parallel determination in our laboratory and in an external reference diagnostic laboratory (Central Diagnostic Laboratory, Medical Center, University of Freiburg). A comparison of the experimental results and those obtained using a validated, automated kinetic Jaffe method are provided in Figure 5a and Table 5 and in the Bland-Altman plot shown in Figure 5b. As shown in Figure 5c, all thiol and thioether metabolites examined in this study were found in detectable amounts in urine. Thus, in the absence of plasma samples, our LC-MS/MS platform could assist in the diagnosis of genetic disorders that affect thiol and thioether metabolites as well as the severe nutritional deficiencies of vitamin B6, B9 and B12 that result in a similar metabolomic signature.

### 4.9. Determination of Thiol and Thioether Metabolites in RBCs: Effect of Sample Handling on Thiol and Thioether Metabolites

**Figure 6:**
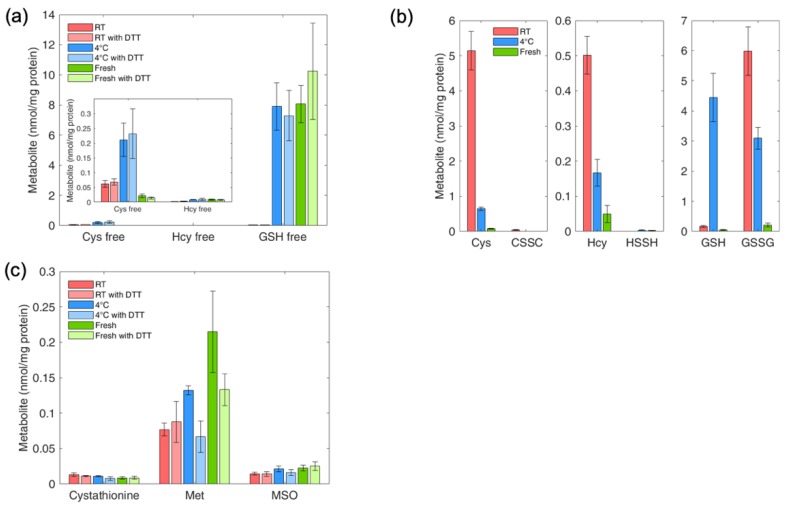
Effect of sample storage conditions on the thiol and thioether metabolite profile of human red blood cells. Blood was drawn from a healthy subject and processed immediately (Fresh) or stored at room temperature (RT) or 4 °C (4 °C) for 16 h for prior to metabolite profiling. Panel (**a**). Pools of free reduced thiols blocked with iodoacetamide, with and without sample treatment with DTT. Panel (**b**). Pools of oxidized and total reduced thiols under the three different sample handling conditions. Panel (**c**). Quantification of thioethers cystathionine, methionine and methionine sulfoxide under the three different sample handling conditions. Values are expressed as the mean ± standard deviation.

Experimental conditions for the measurement of thiol and thioether metabolites in cells were first established in human red blood cells isolated from freshly-drawn blood or after a 24 °C incubation at room temperature or 4 °C. Results of free reduced thiol pools, free reduced and free oxidized pools of Hcy, Cys and GSH, and Cysta, Met and MSO are provided in Figure 6a–c, respectively. Blood storage conditions had a marked effect on the profile of free reduced thiols (Figure 6a) and total reduced and total free oxidized thiols (Figure 6b), but was without effect on the non-redox labile metabolites Cysta, Met and MSO (Figure 6c). In particular, incubation of blood at room temperature led to a nearly complete disappearance of free reduced GSH, compared to the levels found in freshly isolated cells or in those maintained at 4 °C. Free reduced Cys showed maximal concentration after a 24-hour incubation at 4 °C, followed by room temperature and fresh sample preparation, suggesting that the 4 °C read out may represent an overestimation of the concentration of this metabolite. Free reduced Hcy was lowest after a 24-hour room temperature incubation and comparable when the sample was stored for 24 h at 4 °C or processed freshly after blood draw. Analysis of total reduced and total oxidized thiol pools showed that glutathione is the most sensitive metabolite toward storage conditions, with a marked increase in GSSG and a decrease in GSH after a 24-hour room temperature blood incubation. Altogether, these findings suggest that a cohort-wide examination of redox-labile thiol and thioether metabolites would require strict harmonization of sample storage conditions in order to perform a meaningful profiling of the thiol pools. Ideally, on-site treatment of freshly collected blood with APS to obtain APS-plasma would be preferred.

### 4.10. Determination of Thiol and Thioether Metabolites in Various Cell Types

Based on the findings of metabolomic profiling in RBCs, after unifying the post-harvesting sample workup, thiol and thioether metabolites were examined in a variety of cells types. Our results showed that the relative proportion of metabolites appears to be cell-specific (Table 6). The growth of cells in culture medium represents an unnatural condition, and therefore, it is plausible that the composition of the culture medium itself could influence the intracellular and extracellular steady-state concentrations of thiol and thioether metabolites. To test this possibility, two different cell types were grown in parallel in the same culture medium (HepG2 and bovine aortic endothelial cells grown in DMEM 10% FBS). For the metabolites investigated in this study, same culture conditions did not result in equal intracellular content of thiol and thioether metabolites, suggesting that speciation and relative abundance are more strongly influenced by cell type than by nutritional composition of the culture medium. Indeed, the metabolic profile of HepG2 cells more closely resembled the metabolite features of other cancer cells (Table 6, pancreatic cancer cell lines), including high concentrations of total Hcy, Cys, GSH and Met. Pancreatic cancer cells exhibited a higher concentration of MSO compared to all other cell types.

### 4.11. Determination of Thiol and Thioether Metabolites in Conditioned Culture Medium

**Figure 7:**
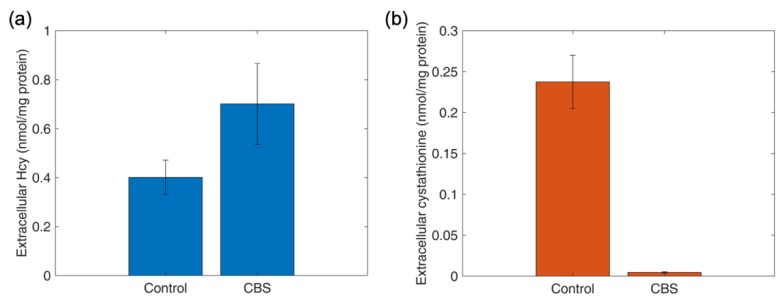
Analysis of marker thiol and thioether metabolites in cystathionine β-synthase deficiency. Cystathionine β-synthase catalyzes the conversion of serine and homocysteine into cystathionine. Panel (**a**). Export of reaction co-substrate Hcy into conditioned culture medium after 7 days by a control subject and a B6-responsive CBS-deficient patient. Panel (**b**). Export of transsulfuration reaction product Cysta into conditioned culture medium after 7 days by a control subject and the B6-responsive CBS-deficient patient.

Cultured cells take up and export a number of nutrients and products of metabolism. Metabolites of the methionine, folate and transsulfuration pathway are no exception. To test whether our targeted metabolomics platform could detect disease-relevant fingerprints, the conditioned culture media of skin fibroblasts from healthy individuals and from a patient with cystathionine β-synthase deficiency, the cause of classic homocystinuria, were compared. As shown in Figure 7, cells from the CBS-deficient patient exported greater amounts of substrate Hcy compared to controls (Figure 7a) and showed essentially no export of the transsulfuration reaction product Cysta (Figure 7b). These metabolite signatures are consistent with loss of CBS enzymatic activity, which catalyzes the biosynthesis of cystathionine from substrates homocysteine and serine.

### 4.12. Determination of Intracellular and Extracellular Thiols and Thioethers in HepG2 Cells upon Oxidant Challenge and Antioxidant Supplementation

**Figure 8:**
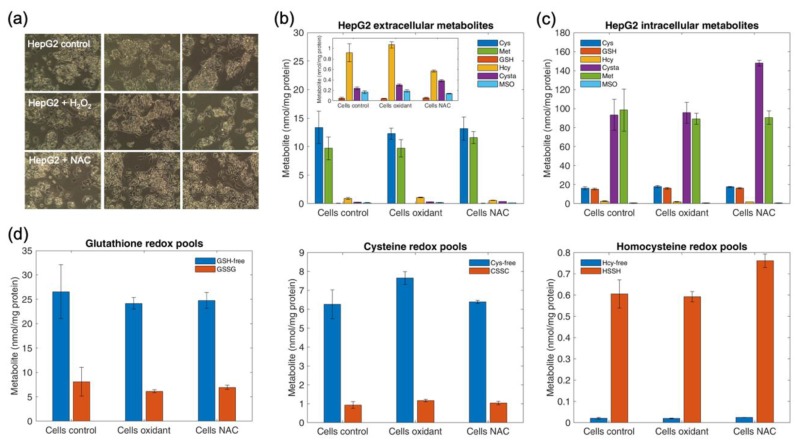
Thiol and thioether profiles in HepG2 cells treated with hydrogen peroxide or *N*-acetylcysteine. HepG cells were grown in normal culture medium (control) or in normal culture medium supplemented with 100 µM hydrogen peroxide (H_2_O_2_, oxidant) or 1 mM *N*-acetylcysteine (NAC) for 24 h. An aliquot of conditioned culture medium was taken to profile extracellular thiol and thioether metabolites. Cells were harvested and processed with APS to quantitate intracellular thiol and thioether metabolites in their oxidized and redox forms. Panel (**a**). HepG2 cells, triplicates in rows, under the different experimental conditions. Panel (**b**). Extracellular metabolites. Each bar represents the sum of oxidized and protein-bound disulfide metabolites, obtained upon treatment of the sample with DTT. Panel (**c**). Intracellular metabolites. Each bar represents the sum of oxidized and protein-bound disulfide metabolites, obtained upon treatment of the sample with DTT. Panel (**d**). Intracellular pools of free reduced and oxidized cysteine, glutathione and homocysteine. Values are expressed as the mean ± standard deviation.

We examined the intracellular and extracellular sulfur-metabolite pools in HepG2 cells grown under normal conditions, upon challenge with 100 µM H_2_O_2_ or in the presence of 1 mM *N*-acetylcysteine (NAC, antioxidant) supplied in the culture medium for 24 h. These experimental conditions did not influence the morphology of HepG2 cells (Figure 8a). An aliquot of the culture medium was set out for metabolomics analysis after the respective 24-h incubations. Cells were harvested and processed as described in Methods. No differences were documented in extracellular metabolite concentrations between experimental conditions (Figure 8b). Only a slight increase in intracellular Cysta and HSSH was observed for cells treated with *N*-acetylcysteine. Oxidative conditions with H_2_O_2_ as well as antioxidant supplementation with *N*-acetylcysteine had no effect on the intracellular pools of GSH, Cys, GSSG and CSSC (Figure 8c). HepG2 cells, similarly to other cell types shown in Table 6, exhibited a higher concentration of GSH and Cys compared to GSSG and CSSC. The exact opposite was found for redox pair Hcy/HSSH in all cells, except in hRTPCs showing Hcy ≈ HSSH.

## 5. Discussion

The primary objective of this targeted metabolic profiling study was to quantify thiol and thioether metabolites in plasma, urine, cells and culture medium.

The optimization of methods for the profiling of thiol and thioether metabolites has been a matter of research over decades [45,46,47,48]. The quantification of thiol pools is most challenging due to the ease of oxidation to form disulfides and mixed disulfides upon exposure to air, as well as enzymatic degradation as it occurs for plasma glutathione upon blood drawing. Early work by Jones and coworkers solved this issue by preserving the thiol pools prior to chemical derivatization and fluorescence detection [35]. Several methods for the targeted determination of thiol and thioether metabolites were published thereafter, but none have jointly examined thiol pools and key metabolites of the methionine, folate and transsulfuration pathways applicable to various biological matrices. In addition, very few studies have provided validation with standardized external quality controls or cohort analysis versus established reference ranges.

Metabolite extraction was performed with aqueous acidic methanol (final concentration 60% MeOH, pH 2.2), which was suitable for downstream chromatographic separation and ionization. Despite the polarity of the species examined in this study, good column retention under reversed phase conditions without detectable carry over were observed. Extraction of metabolites with methanol/water mixes in various proportions has been widely used in targeted and untargeted metabolomic approaches [41,49,50,51,52]. Matrix effect was observed mainly for Hcy and Cys. To our knowledge, matrix effects can be dealt with in two major ways: (a) optimizing sample preparation to eliminate interfering agents, or (b) combining a robust sample preparation protocol with the use of isotopically labeled standards. Our method utilized solvents compatible with mass spectrometry that minimized the occurrence of ion suppression or enhancement effects and we also used stable isotopically labeled standards to account for matrix effects. Our calibration curves were made in water. An identical matrix devoid of the target metabolite is the optimal solvent for the preparation of calibrators, yet this is not readily available. The use of biological matrix as a solvent for the calibration curves could introduce error because the metabolites of interest are endogenous components of mammalian fluids and cells. Using commercial plasma, urine or cell lysates as reference matrices requires quantifying the endogenous concentration of those metabolites from lot to lot, which can be impractical. In the case of urinary creatinine, using urine as the biological matrix is simply not possible, due to the very high concentration of this metabolite in this biological fluid. Our assessment of whether the preparation of calibrators in water or in the respective biological matrices had a significant effect in the calculated concentration of metabolites suggests that under our experimental conditions, the use of calibrators prepared in water together with the use of isotopically labeled internal standards provides sufficient accuracy and reproducibility both for diagnostic and research purposes.

The majority of mammalian fluids, tissues and cells have a pH of 6–7. A previous study showed that thiols and thioethers are stable over a broad range of pHs [22]. More generally, polar metabolites exhibit relatively good stability under harmonized long-term storage conditions [53,54]. Plasma isolated from EDTA-preserved blood has proven suitable for quantitative metabolomics [41,53,54] and this was the blood preservation vehicle chosen in this study. The determination of total metabolite concentrations in plasma as required for diagnostic purposes was carried out in the presence of excess DTT to reduce free disulfides and protein-bound disulfides. The concentration of free reduced thiol pools of Hcy, Cys and GSH was made possible via derivatization of the respective thiol groups with iodoacetamide, which halts thiol oxidation during sample preparation. The concentration of free reduced, free oxidized and total thiol and thioethers determined by our method agree with those published and reviewed by independent research groups [2,22]. For established biomarkers, Hcy, Cys, Met, Cysta and Crea, the total concentrations determined with the method in this study are within the reference ranges for healthy human subjects.

Our method is fast, inexpensive, requires minimal sample processing and showed reliable performance examined by reference quality controls, determination of plasma metabolites in various animal species, and testing in a cohort of 53 healthy human subjects. The high sensitivity of this method permitted the quantification of 11 metabolites in only 20–40 µL of plasma and with a LC-MS/MS running time of 5 min. Under these experimental conditions, 80 samples per day could be processed and run. Total sample preparation volumes of this method for all matrices (160 µL) are suitable to operate on automated metabolomic platforms, enabling the processing of even greater number of samples per day. The inclusion of creatinine into the panel of metabolites permitted the normalization of metabolite concentrations in urine under the same sample preparation protocol and LC-MS/MS conditions, thus eliminating the need for third-party sample processing and variations associated to the quantification using different methods. Concerning plasma, work by Ulvik et al. recently highlighted greater sensitivity and specificity of metabolite ratios Hcy:Cys and Hcy:Cys:Crea compared to Hcy alone for the assessment of B-vitamin status [18]. Our LC-MS/MS platform permits the simultaneous determination of Hcy, Cys and Crea for the optimal examination of B-vitamin status in large population studies.

This study revealed that the distribution and redox state of thiol and thioether metabolites is specific to cell types. As expected, analysis of Cys/CSSC and GSH/GSSG showed greater concentration of the reduced species over the oxidized species in all cell types. The exact opposite relationship was found for Hcy/HSSH, except in hRTPCs where Hcy ≈ HSSH. It remains to be elucidated whether this distinct Hcy/HSSH poise holds relevance for renal physiology. Importantly, we found that processing conditions of cells prior to analysis strongly influences the relative concentration of redox active species, requiring that storage and transportation are consistent across samples if metabolomic comparisons are intended.

Treatment of HepG2 cells with the oxidant H_2_O_2_ or the antioxidant *N*-acetylcysteine was without effect on thiol and thioether metabolites, which is in accord with previous work demonstrating that cancer cells are exquisitely resistant to oxidative stress [55,56] and strongly reliant on methionine metabolism [20]. HepG2 cells exhibited a substantially higher concentration of intracellular Met compared to all other cell types, a metabolic feature that was shared with three lines of pancreatic cancer cells.

Analysis of extracellular medium has been of great value in the field of inherited metabolic diseases to study the phenotype of cultured cells with respect to the disease of interest [37,57,58,59,60,61,62]. Accumulation of substrates and products resulting from impaired biochemical pathways are often reflected in conditioned culture medium as much as they appear in the plasma of diseased individuals. The determination of total metabolites in conditioned culture medium was performed with high sensitivity, permitting the identification of disease-specific metabolic features of CBS deficiency, namely, low Cysta and elevated Hcy, the hallmark metabolite fingerprints established since early discovery and characterizations of the disease [63].

Our LC-MS/MS platform is suitable to perform studies where intra-individual paired samples may be of relevance, for example, to reconstruct metabolic pathways and to disentangle organ-specific features from systemic or constitutive features. The protocols established for cells and culture medium open possibilities beyond comparisons between genotypes and diseases. Our protocols are suitable for metabolic labeling studies to assess flux through methionine, folate and transsulfuration metabolism, to quantitatively determine the effect of nutrient deficiencies and the response to pharmacological interventions in vitro.

## 6. Strengths and Limitations of the Study

Among the strengths of the study are the use of low sample volume (20–40 µL plasma or urine or 500,000 cells), simple sample preparation protocols, short running time (5 min) and open validation with commercial internal standards and quality controls that are available to all researchers. Quantification of the urinary biomarker creatinine was validated by comparison with an external laboratory that utilized a standardized automated kinetic Jaffe method. Sample preparation protocols were harmonized for plasma, urine, cells and conditioned culture medium, to a total volume of 160 µL enabling method transfer into automated metabolomic platforms.

Limitations of the study include the need to instruct medical personnel on the preservation of blood samples on site for the analysis of oxidized and reduced thiols. For cells, debate exists on which is the best procedure to minimize artifactual metabolite readouts caused by cell harvesting techniques. Our study only evaluated trypsinization, as this is the method widely used for the examination of enzymatic activities, protein expression and nucleic acid profiling. This approach permits metabolomic analysis while sparing cell lysate under near native conditions that could be utilized to examine other cellular parameters on the exact same sample. Other protocols, especially in untargeted metabolomics, employ metabolic quenching with methanol followed by cell scrapping and direct metabolite extraction. While this appears to minimize the loss of signal intensity for some metabolites, such sample treatment precludes concerted sample use for other experimental purposes. While our method quantitates homocysteine, a key biomarker of folate status, it does not determine folate metabolites. An elegant study is available for the determination of folate species in various biological matrices [64]. Finally, our study examined intracellular and extracellular thiol and thioether metabolites in cells grown in standard culture media but did not examine metabolite profiles in nutrient-deprived culture media that may be relevant for disease modeling or nutrition studies. This application is currently underway in our laboratory.

## 7. Conclusions

Our LC-MS/MS platform permits the quantitative determination of 11 metabolites in plasma, urine, cells and culture medium in a running time of 5 min. Low limit of quantification enabled the use of 20–40 µL of plasma and 250,000–500,000 cells as starting material. Quality controls and validation in a cohort of 53 healthy subjects as well as in commercially available animal plasma yielded results that are in accord with reference ranges established by accredited diagnostic laboratories and literature from different research groups. The method was extended with success to determine thiol and thioether metabolites and creatinine in urine, cells and conditioned culture medium. The ease of sample preparation and short running time should facilitate high-throughput analysis of specimens relevant to health and disease.

## Figures and Tables

**Table 1:** Mass transitions and optimized mass spectrometry parameters for the detection of thiol and thioether metabolites and creatinine.

Metabolite	Abbreviated ID	Retention Time (min)	Q1 Mass (Da)	Q3 Mass (Da)	Dwell Time (ms)	DP (Volts)	EP (Volts)	CE (Volts)	CXP (Volts)
Homocysteine	Hcy	1.47	135.976	90	20	31	10	19	10
Homocystine	HSSH	1.32	268.978	136.004	20	1	10	13	6
Cysteine	Cys	1.36	121.984	76	20	31	10	17	10
Cystine	CSSC	1.24	240.98	151.905	20	26	10	17	4
Oxidized glutathione	GSSG	2.71	612.987	355.072	20	86	10	31	18
Reduced glutathione	GSH	2.25	307.979	179	20	1	10	15	8
*S*-methylglutathione	GSMe	3.32	322.035	176.082	20	46	10	21	18
Cystathionine	Cysta	1.18	223.1	134	20	26	10	19	16
Methionine	Met	1.65, 1.82	149.987	103.9	20	56	10	17	12
Methionine sulfoxide	MSO	1.36	166.01	74.1	20	1	10	19	6
Cysteine blocked	Cys-free	1.36	179	162.1	20	100	10	10	10
Glutathione blocked	GSH-free	1.91	365	236.1	20	100	10	10	10
Homocysteine blocked	Hcy-free	1.38	193	147.2	20	100	10	10	10
Creatinine	Crea	0.90	114.023	43.9	20	10	10	27	8
D3-creatinine	D3-Crea	0.90	117.094	47	20	10	10	27	8
D4-homocysteine	D4-Hcy	1.44	139.976	94	20	31	10	19	10
D4-cysteine	D4-Cys	1.35	125.984	79	20	31	10	17	10
^13^C_2_,^15^N glutathione (reduced)	^13^C_2_,^15^N-GSH	2.23	310.979	179	20	46	10	15	8
D4-cystathionine	D4-Cysta	1.16	227.1	138	20	26	10	19	16
D4-methionine	D4-Met	1.63, 1.79	153.987	107.9	20	56	10	17	12

**Table 2:** Linearity, limit of quantification and inter-assay variation.

Metabolite ^(a)^	Linearity (µmol/L)	LoQ (µmol/L)	Inter-assay Variation (%)
Cysteine	0–300	0.9	2.95
Cystine	0–100	0.04	11.22
Homocysteine	0–100	0.35	4.86
Homocystine	0–50	0.02	10.2
Glutathione	0–20	0.05	2.56
Glutathione disulfide	0–20	0.63	7.57
Methionine	0–150	1.65	3.74
Methionine sulfoxide	0–150	0.08	3.73
Cystathionine	0–50	0.01	2.64
Creatinine	0–500	0.01	2.35

^(a)^ Linearity, limit of quantification and inter-assay variation for derivatized versions of Cys, Hcy and GSH did not differ from those of their corresponding parent metabolite.

**Table 3:** Thiol and thioether metabolites and creatinine in commercial human and animal plasma.

Metabolite (µmol/L)							
Sample	Creatinine	Cystathionine	Cysteine	GSH	Homocysteine	Methionine	MSO
Mouse 1	6.7 ± 1.0	0.701 ± 0.098	148 ± 13	71 ± 6	4.0 ± 0.5	55 ± 5	0.74 ± 0.08
Mouse 2	7.9 ± 0.7	0.783 ± 0.130	139 ± 18	79 ± 3	4.2 ± 0.3	56 ± 3	0.81 ± 0.04
Human	69 ± 13	0.115 ± 0.010	181 ± 9	3.7 ± 0.5	7.5 ± 0.7	25 ± 1	2.21 ± 0.34
Dawley Rat	20 ± 4	0.443 ± 0.070	163 ± 12	29 ± 3	4.1 ± 0.2	60 ± 3	0.90 ± 0.07
Beagle Dog	55 ± 5	2.014 ± 0.150	128 ± 17	11.7 ± 0.6	7.4 ± 0.5	50 ± 3	1.07 ± 0.11
QC	74 ± 6	0.275 ± 0.040	269 ± 25	2.6 ± 0.3	57 ± 3	29 ± 2	0.70 ± 0.09
**Human**	69 ± 13	0.115 ± 0.01	181 ± 9	3.7 ± 0.5	7.5 ± 0.7	24 ± 2	2.21 ± 0.34
**Reference Range**	< 90	< 0.400	150-350	2–5.1	5–15	18–33	N/A ^(a)^
QC Hcy Expected	44–65	-	-	-	-	-	-
QC Hcy Experimental	57 ± 3	-	-	-	-	-	-

^(a)^ In a study of 8 healthy human subjects, a reference value of 4.0 ± 1.0 µM was established (Analytical Biochemistry, Volume 313, Issue 1, 1 February 2003, Pages 28–33). The authors stated that MSO amounts to approximately 10% of the concentration of Met.

**Table 4:** Concentration of thiol and thioether metabolites in healthy individuals (*n* = 11).

	Metabolite (µmol/L)											
Subject ID	Cys-Free	GSH-Free	Hcy-Free	Cysta	Cys	CSSC	GSH	GSSG	Hcy	HSSH	Met	MSO
Subject 1	7.4	3.6	0.11	0.42	248	54	6.4	0.3	6.9	0.045	32	1.0
Subject 2	6.1	3.7	0.14	0.14	264	54	10.1	1.8	8.1	0.019	30	0.7
Subject 3	6.0	2.6	0.10	0.18	312	59	7.5	4.0	12.8	0.029	28	0.9
Subject 4	5.9	2.4	0.17	0.15	275	49	8.6	3.9	13.5	0.030	32	1.2
Subject 5	5.9	2.2	0.11	0.17	237	48	7.0	4.1	9.4	0.021	40	1.0
Subject 6	7.4	1.6	0.15	0.15	234	55	7.4	3.9	6.1	0.021	48	1.8
Subject 7	6.8	1.7	0.21	0.14	217	46	6.4	3.6	7.9	0.024	34	1.7
Subject 8	7.9	1.5	0.22	0.32	285	63	7.1	4.3	10.8	0.065	44	1.9
Subject 9	5.5	1.7	0.09	0.19	246	52	7.4	4.1	6.9	0.020	34	1.4
Subject 10	4.0	3.6	0.17	0.16	257	53	6.8	4.1	8.4	0.019	26	0.8
Subject 11	4.8	1.9	0.14	0.31	307	68	6.7	3.9	10.7	0.031	33	1.2
Mean	6.2	2.4	0.15	0.21	262	54	7.4	3.5	9.2	0.029	35	1.2
SD	1.2	0.9	0.04	0.09	30	7	1.1	1.2	2.4	0.014	7	0.4
Reference range ^(a)^	8.3–10.7	2.0–5.1	0.17–0.32	<0.4	202–281	41–63	4.9–7.3	0.7–1.6	6.5–11.9	1.0–1.2	18–35	N/A ^(b)^

^(a)^ Reference ranges were taken from Turell et al. (Free Radic. Biol. Med. 2013, 65, 244–253) and from Bevital Laboratories (Bevital AS, Bergen, Norway); ^(b)^ In a study of 8 healthy human subjects, a reference value of 4.0 ± 1.0 µM was established (Analytical Biochemistry, Volume 313, Issue 1, 1 February 2003, Pages 28–33). The authors stated that MSO amounts to approximately 10% of the concentration of Met.

**Table 5:** Creatinine concentration in urine of healthy human subjects (*n* = 5).

Urine Sample ID	Creatinine Kinetic Jaffe Method (mM)	Creatinine LC-MS/MS (mM) ^a^
Subject 1	1.32	1.47
Subject 2	8.25	10.56
Subject 3	10.26	13.06
Subject 4	11.45	15.00
Subject 5	3.42	3.71

^a^ Values are the average of 3 independent experiments.

**Table 6:** Profile of thiol and thioether metabolites in several cell types (nmol/mg protein).

Cell Type	BAEC	NHDF	GM13395	hRTPCs	Panc 05.04	MiaPaCa-2	AsPC-1	HepG2	hESC ^a^
**Genotype**	Normal	Normal	MTHFR	Normal	Cancer	Cancer	Cancer	Cancer	Normal
**Organ**	Endothelium	Skin	Skin	Kidney	Pancreas	Pancreas	Pancreas	Liver	Stem cells
**Cys free**	9.2 ± 1.8	18.6 ± 3.5	2.07 ± 0.45	11.7 ± 3.5	23.1 ± 4.3	26.0 ± 3.3	22.1 ± 3.4	6.26 ± 0.77	-
**Hcy free**	0.14 ± 0.04	0.11 ± 0.06	0.027 ± 0.015	0.15 ± 0.02	0.12 ± 0.05	0.06 ± 0.01	0.05 ± 0.03	0.021 ± 0.004	-
**GSH free**	40.9 ± 3.9	57.2 ± 7.1	5.64 ± 1.59	19.6 ± 10.5	43.0 ± 4.2	68.3 ± 3.1	34.6 ± 1.9	26.5 ± 5.6	-
**CSSC**	1.90 ± 0.51	2.56 ± 1.38	11.6 ± 4.9	0.33 ± 0.25	8.76 ± 0.63	2.90 ± 0.81	6.94 ± 0.50	0.93 ± 0.18	-
**HSSH**	0.18 ± 0.05	0.22 ± 0.03	0.50 ± 0.13	0.11 ± 0.02	5.03 ± 0.25	0.77 ± 0.09	4.35 ± 0.16	0.60 ± 0.07	-
**GSSG**	1.58 ± 0.92	5.18 ± 0.65	12.8 ± 3.1	0.67 ± 0.56	8.61 ± 0.78	18.6 ± 0.81	6.14 ± 0.38	8.09 ± 2.94	-
**Cys**	8.63 ± 2.59	14.2 ± 5.9	46.2 ± 14.2	2.78 ± 1.09	55.3 ± 2.5	30.0 ± 3.3	47.0 ± 4.6	16.1 ± 1.6	7.03 ± 0.40
**Hcy**	0.64 ± 0.11	0.46 ± 0.12	0.53 ± 0.13	0.17 ± 0.03	4.34 ± 0.15	1.84 ± 0.18	4.78 ± 0.54	2.60 ± 0.42	0.017 ± 0.002
**GSH**	1.87 ± 0.67	3.84 ± 0.47	8.41 ± 1.34	0.99 ± 0.51	19.6 ± 3.0	20.0 ± 1.2	17.0 ± 0.3	15.3 ± 0.9	3.96 ± 0.19
**Cysta**	5.56 ± 1.07	4.74 ± 0.68	1.66 ± 0.60	3.17 ± 1.00	2.40 ± 0.34	55.2 ± 8.9	2.83 ± 0.18	93.4 ± 16.4	0.602 ± 0.016
**Met**	62.0 ± 11.3	29.1 ± 5.4	46.4 ± 8.30	11.8 ± 1.6	468 ± 65	193 ± 13	217 ± 4.5	98.4 ± 22.3	4.38 ± 0.10
**MSO**	212 ± 40	184 ± 38	256 ± 49	60.2 ± 7.1	1614 ± 165	377 ± 15	873 ± 50	0.78 ± 0.13	0.056 ± 0.003

^a^ Due to a ban of possession of intact human embryonic stem cells in Germany, only total concentrations of metabolites were determined in hESC.

## Supplementary Materials

The following are available online at https://www.mdpi.com/2218-1989/9/10/235/s1. Figure S1. Sample preparation workflows. Panel (a). Sample preparation for APS-plasma, required for the profiling of oxidized and reduced thiol species in plasma. Panel (b). Sample preparation with APS for cell lysates, required for the profiling of intracellular reduced and oxidized thiol species; Figure S2. Sample preparation workflows. Panel (a). Sample preparation for urine. This method quantifies total thiol and thioether metabolites and creatinine. Panel (b). Sample preparation for conditioned culture medium. This method quantifies total thiol and thioether metabolites and creatinine; Figure S3. Sample preparation workflows. Panel (a). Sample preparation for untreated plasma, the starting specimen typically used in clinical diagnostic laboratories. This method quantifies total thiol and thioether metabolites and creatinine; Figure S4. Calibration curves of the metabolites examined in this study. A linear fit forced to intercept at x = 0, y = 0 was applied in all cases. All curves consist of 9–12 calibration points, using pure commercially available substances; Figure S5. Effect of sample matrix on the quantification of thiol and thioether metabolites with diagnostic value. Panel (a). Calibration curves made in water as solvent or in extracted plasma, cell lysate and urine. Panel (b). Peak area of isotopically labelled internal standards D3-creatinine, D4-homocysteine, D4-cysteine, D4-methionine, D4-cystathionine and ^15^N, ^13^C_2_-glutathione compared to GSMe in calibrators (blue), quality control (lyophilized serum, orange) and human plasma (yellow); Figure S6. Pearson’s correlation among thiol and thioether metabolites examined in this study. Pearson’s coefficients are given in the upper left corner of each graph. No correlations were observed at a significance level of 0.05; Table S1. Stability of thiol and thioether metabolites in human plasma; Table S2. Slopes and goodness of linear fits (R2) from calibration curves made with different biological matrices; Table S3. Concentration of Hcy in quality control and commercial pooled human plasma determined with calibration curves prepared with various biological matrices; Table S4. Analysis of thiols and thioethers in untreated plasma as typically processed in diagnostic laboratories, in a cohort of healthy individuals (N = 53).

## Abbreviations

LC-MS/MS: liquid chromatography tandem mass spectrometry; Hcy: homocysteine; HSSH: homocystine, oxidized homocysteine; tHcy: total plasma Hcy as measured in diagnostic laboratories; Cys: cysteine; CSSC: cystine, cysteine oxidized; GSH: glutathione; GSSG: glutathione oxidized; Met: methionine; MSO: methionine sulfoxide; Cysta: cystathionine; Crea: creatinine; Hcy-blocked: pool of free reduced Hcy, blocked with iodoacetamide; Cys-blocked: pool of free reduced Cys, blocked with iodoacetamide; GSH-blocked: pool of free reduced GSH, blocked with iodoacetamide; GSMe: *S*-methylglutathione; NAC: *N*-acetylcysteine; IS: internal standard; Ser: serine; MTHFR: methylentetrahydrofolate reductase; MMACHC/cblC: methylmalonic aciduria combined with homocystinuria type C; CBS: cystathionine β-synthase; ERNDIM: European Research Network for evaluation and improvement of screening, Diagnosis and treatment of Inherited disorders of Metabolism.
