# Supplementary material for: Targeted Metabolic Profiling of Methionine Cycle Metabolites and Redox Thiol Pools in Mammalian Plasma, Cells and Urine
Source: Metabolites. 2019 Oct 18;9(10):235. doi: 10.3390/metabo9100235 (PMC6836102; doi:10.3390/metabo9100235)
Supplement: Supplementary file 1 [file metabolites-09-00235-s001.pdf]

Supplemental Material for

Targeted metabolic profiling of methionine cycle metabolites and redox thiol pools in mammalian plasma, cells and urine.

Sidney Behringer[1], Victoria Wingert[1], Victor Oria[2,3], Anke Schumann[1,4], Sarah Grünert[4], Artur Cieslar-Pobuda[5], Stefan Kölker[6], Ann-Kathrin Lederer[7], Donald W. Jacobsen[8], Judith Staerk[5,9], Oliver Schilling[2], Ute Spiekerkoetter[4] and Luciana Hannibal[1*]

[1]Laboratory of Clinical Biochemistry and Metabolism, Department of General Pediatrics, Adolescent Medicine and Neonatology, Medical Center, University of Freiburg, Freiburg, Germany.

[2]Institute of Surgical Pathology, Medical Center - University of Freiburg, Freiburg, Germany.

[3]Spemann Graduate School of Biology and Medicine, Faculty of Biology, University of Freiburg, Freiburg, Germany.

[4]Department of General Pediatrics, Adolescent Medicine and Neonatology, Medical Center, University of Freiburg, Freiburg, Germany.

[5]Nordic European Molecular Laboratory (EMBL) Partnership, Centre for Molecular Medicine Norway, University of Oslo and Oslo University Hospital, Oslo, Norway.

[6]Centre for Child and Adolescent Medicine, Department of General Pediatrics, Division of Neuropediatrics and Metabolic Medicine, University Hospital Heidelberg, Heidelberg, Germany.

[7]Center for Complementary Medicine, Institute for Infection Prevention and Hospital Epidemiology, Medical Center – University of Freiburg, Faculty of Medicine, University of Freiburg, Freiburg, Germany.

[8]Department of Cardiovascular and Metabolic Sciences, Lerner Research Institute, Cleveland Clinic, Cleveland, USA.

[9]Norwegian Center for Stem Cell Research, Department of Immunology, Oslo University Hospital, 0372 Oslo, Norway

*Correspondence: luciana.hannibal@uniklinik-freiburg.de; Tel.: +49-761-270-43710

Figures

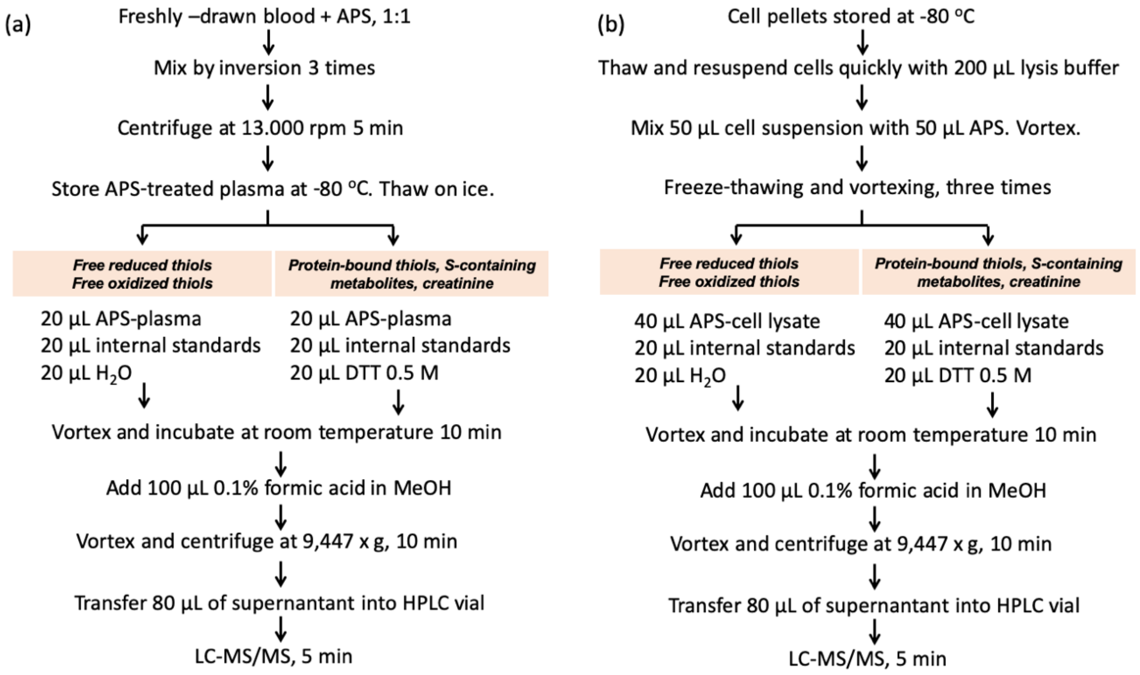

Figure S1. Sample preparation workflows. Panel A. Sample preparation for APS-plasma, required for the profiling of oxidized and reduced thiol species in plasma. Panel B. Sample preparation with APS for cell lysates, required for the profiling of intracellular reduced and oxidized thiol species.

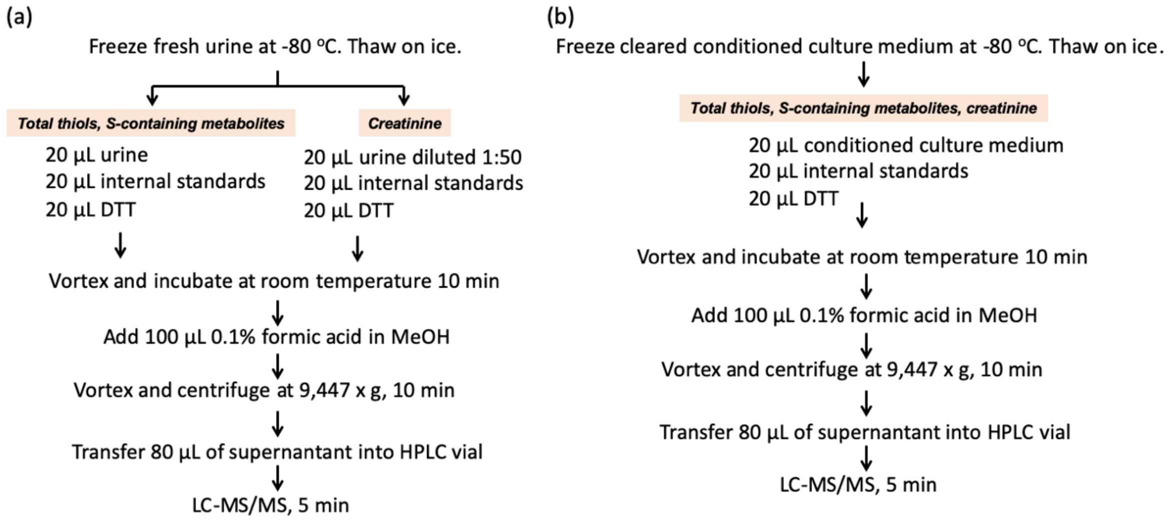

Figure S2. Sample preparation workflows. Panel A. Sample preparation for urine. This method quantifies total thiol and thioether metabolites and creatinine. Panel B. Sample preparation for conditioned culture medium. This method quantifies total thiol and thioether metabolites and creatinine.

(a)

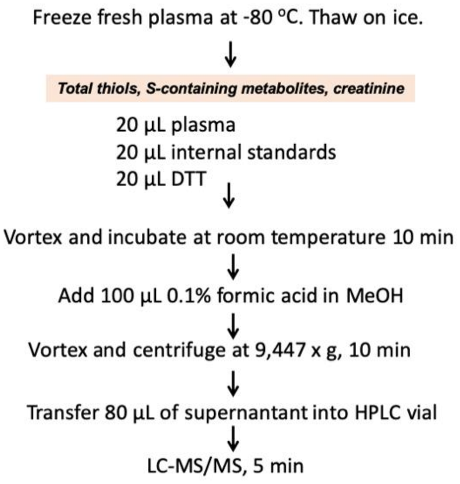

Figure S3. Sample preparation workflows. Panel A. Sample preparation for untreated plasma, the starting specimen typically used in clinical diagnostic laboratories. This method quantifies total thiol and thioether metabolites and creatinine.

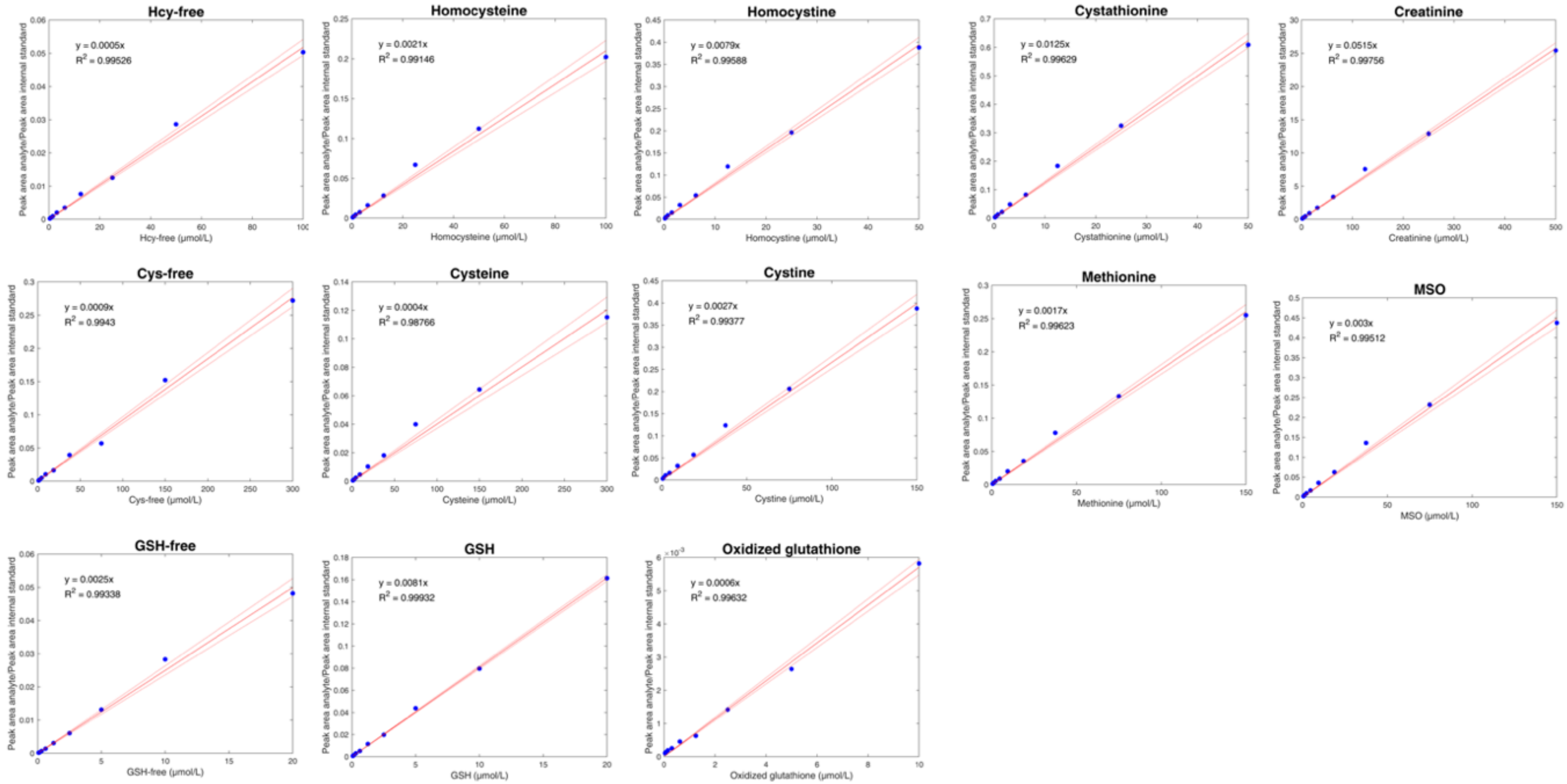

Figure S4. Calibration curves of the metabolites examined in this study. A linear fit forced to intercept at x = 0, y = 0 was applied in all cases. All curves consist of 9–12 calibration points, using pure commercially available substances.

(a)

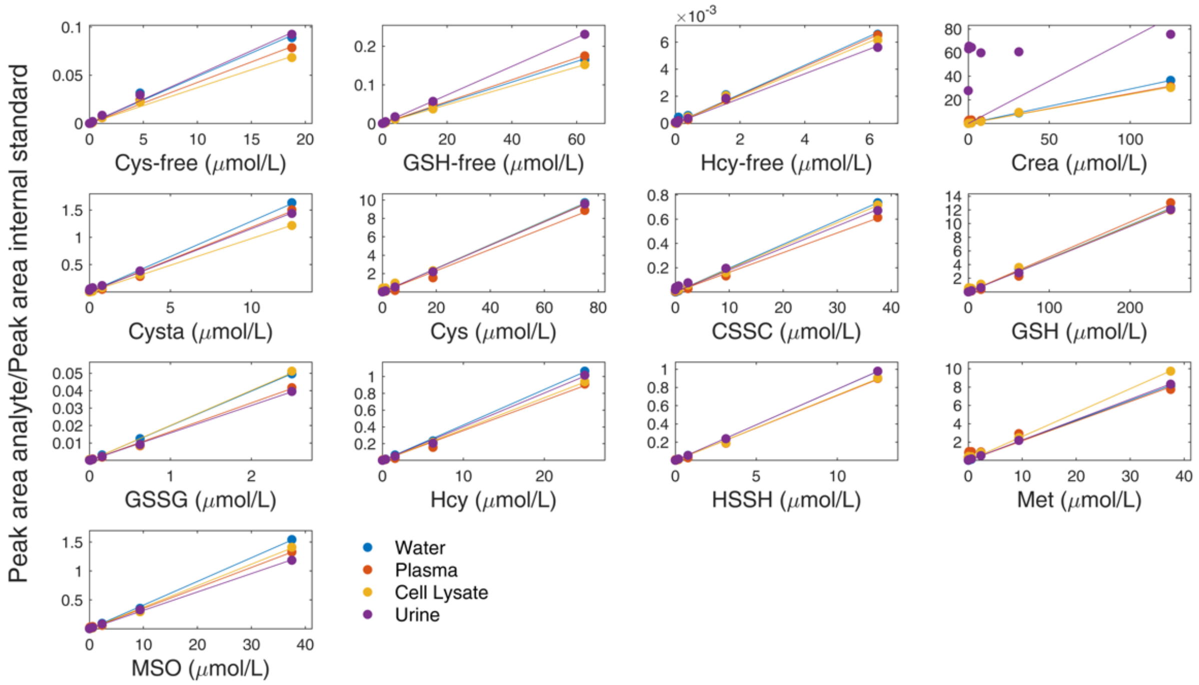

(b)

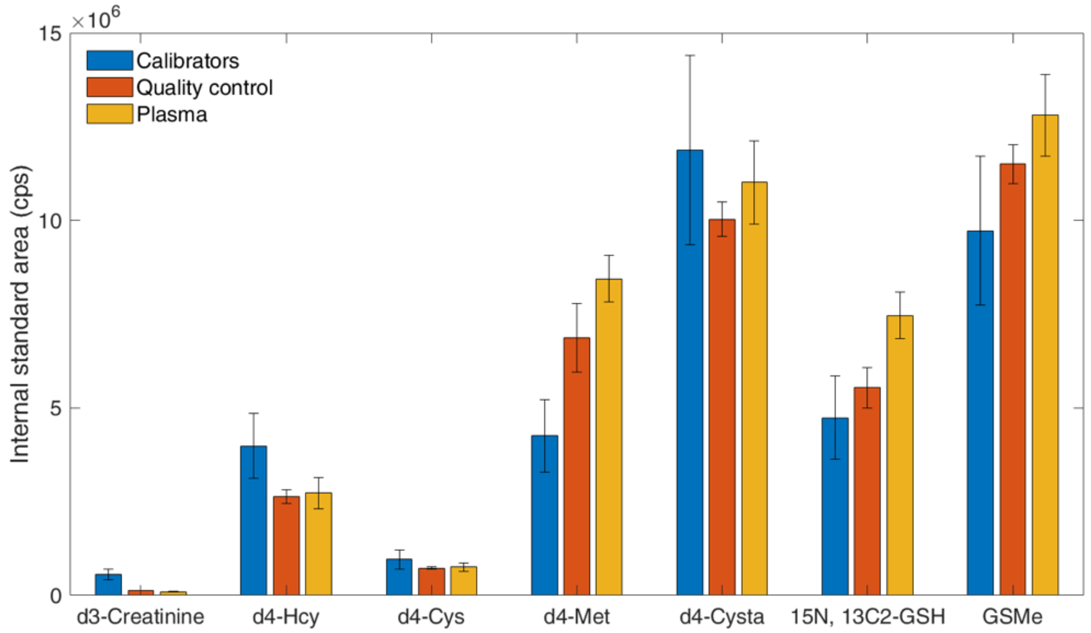

Figure S5. Effect of sample matrix on the quantification of thiol and thioether metabolites with diagnostic value. Panel (**a**). Calibration curves made in water or in extracted plasma, cell lysate and urine as solvent. With the exception of the calibration curve of creatinine made in urine as the biological matrix, all other metabolites exhibited linear trends that did not deviate markedly from that of the curve made with water as the solvent. Slopes and goodness of fit (R^2) for all metabolites are provided in Table S4. Panel (**b**). Peak area of isotopically labelled internal standards D3-creatinine, D4-homocysteine, D4-cysteine, d4-methionine, D4-cystathionine and ^{15}N, $^{13}C_2$-glutathione compared to GSMe in calibrators (blue), quality control (lyophilized serum, orange) and human plasma (yellow).

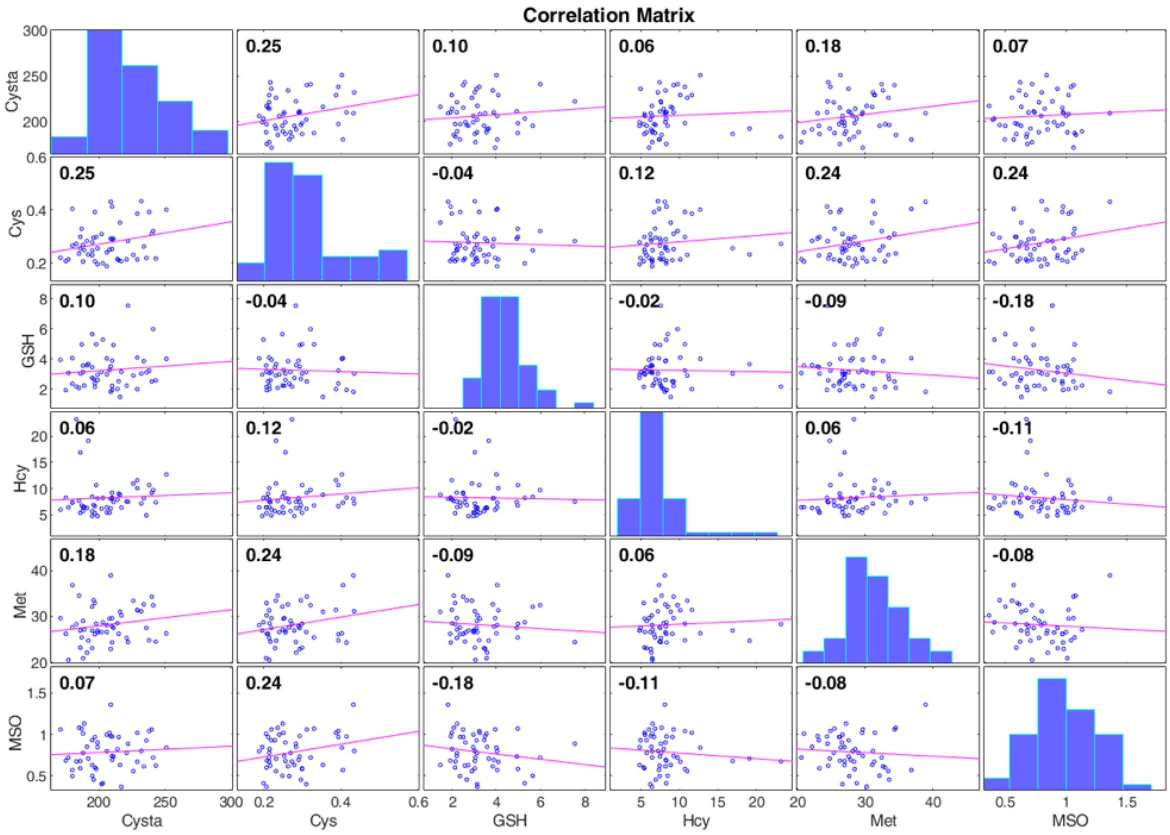

Figure S6. Pearson's correlation among thiol and thioether metabolites examined in this study. Pearson's coefficients are given in the upper left corner of each graph. No correlations were observed at a significant level of 0.05.

Tables

Table S1. Stability of thiol and thioether metabolites in human plasma.

		Metabolite (µmol/L)								
**Sample**	**Time (min)**	**Cysta**	**Cys**	**CSSC**	**GSH**	**GSSG**	**Hcy**	**HSSH**	**Met**	**MSO**
Subject 1	0	0.544	7.9	72.4	1.4	0.72	0.469	0.151	21.7	1.56
	15	0.53	5.83	72.2	0.844	0.64	0.41	0.151	20.1	1.62
	30	0.511	2.73	63	0.586	0.632	0.514	0.139	19.1	1.58
	60	0.514	3.81	66.8	0.61	0.454	0.522	0.145	18.5	1.56
Subject 2	0	0.398	8.95	64.5	1.66	0.487	0.522	0.185	25.4	1.57
	15	0.398	5.46	62.5	1.08	0.447	0.581	0.183	25.3	1.59
	30	0.402	3.37	67	0.893	0.458	0.592	0.175	27.8	1.6
	60	0.394	2.34	54.4	0.673	0.865	0.592	0.145	26	1.6
Subject 3	0	0.401	8.06	77.4	1.87	0.354	0.439	0.16	25.5	1.34
	15	0.401	6.74	74.1	1.28	0.226	0.417	0.157	25.8	1.36
	30	0.403	4.2	69.2	0.912	0.22	0.573	0.147	24	1.34
	60	0.402	5.73	78.9	1.08	0.257	0.607	0.156	26.2	1.36
Subject 4	0	0.555	11.3	72.6	1.93	0.259	0.521	0.167	30.7	1.61
	15	0.562	8.53	72.6	1.09	0.253	0.452	0.164	29.3	1.61
	30	0.553	6.02	70.7	0.741	0.185	0.692	0.157	29.3	1.62
	60	0.56	4.6	68.5	0.68	0.168	0.633	0.147	30.2	1.63

Table S2. Slopes and goodness of linear fits (R^2) from calibration curves made with different biological matrices.

Slopes (y = ax; where a is the slope from linear fits forced to intercept at 0,0)													
**-**	**Cys-free**	**GSH-free**	**Hcy-free**	**Crea**	**Cysta**	**Cys**	**CSSC**	**GSH**	**GSSG**	**Hcy**	**HSSH**	**Met**	**MSO**
**Water**	0.0049	0.0027	0.0011	0.2919	0.1294	0.1289	0.0196	0.0490	0.0199	0.0423	0.0780	0.2179	0.0410
**Plasma**	0.0042	0.0028	0.0010	0.2536	0.1184	0.1159	0.0162	0.0512	0.0165	0.0357	0.0711	0.2138	0.0354
**Cell Lysate**	0.0037	0.0024	0.0010	0.2466	0.0977	0.1281	0.0190	0.0486	0.0201	0.0374	0.0717	0.2605	0.0372
**Urine**	0.0050	0.0037	0.0009	0.7189	0.1158	0.1271	0.0182	0.0479	0.0158	0.0402	0.0782	0.2225	0.0318
**R^2**	-	-	-	-	-	-	-	-	-	-	-	-	-
**-**	**Cys-free**	**GSH-free**	**Hcy-free**	**Crea**	**Cysta**	**Cys**	**CSSC**	**GSH**	**GSSG**	**Hcy**	**HSSH**	**Met**	**MSO**
**Water**	0.987	0.997	0.990	1.000	0.998	1.000	1.000	0.999	1.000	0.999	1.000	0.999	1.000
**Plasma**	0.996	0.998	0.999	0.971	0.994	0.991	0.998	0.992	0.997	0.991	0.997	0.940	0.998
**Cell Lysate**	0.993	1.000	0.992	0.996	1.000	0.994	0.998	0.991	0.992	0.999	0.998	0.995	0.998
**Urine**	0.993	1.000	0.992	0.522	0.994	0.999	0.985	1.000	0.999	0.997	1.000	1.000	0.999

Table S3. Concentration of Hcy in quality control and commercial pooled human plasma determined with calibration curves prepared with various biological matrices.

**Hcy (µmol/L)**	**Water**	**Plasma**	**Cell lysate**	**Urine**
QC1	46	54	51	48
QC2	48	56	54	50
QC3	45	54	51	48
Mean	46	55	52	49
Standard deviation	1	2	1	1
Human plasma 1	6.8	8.0	7.6	7.1
Human plasma 2	6.1	7.2	6.9	6.4
Human plasma 3	7.5	8.8	8.4	7.9
Human plasma 4	8.7	10.3	9.8	9.2
Human plasma 5	7.0	8.2	7.9	7.3
Mean	7.2	8.5	8.1	7.6
Standard deviation	1.0	1.2	1.1	1.0

Table S4. Analysis of thiols and thioethers in untreated plasma as typically processed in diagnostic laboratories, in a cohort of healthy individuals (N= 53).

	**Metabolite (µmol/L)**					
**Sample ID**	**Cysta**	**Cys**	**GSH**	**Hcy**	Met	MSO
Subject 1	0.393	238	3.23	10.7	24.8	1.04
Subject 2	0.299	186	3.03	5.43	23.2	0.59
Subject 3	0.188	206	4.04	6.44	28.1	0.795
Subject 4	0.251	208	3.26	5.44	29.5	1.02
Subject 5	0.402	251	4.01	12.7	26.3	0.839
Subject 6	0.414	210	1.94	8.32	24.3	0.975
Subject 7	0.196	199	2.96	4.82	26.9	0.845
Subject 8	0.214	229	4.09	10.1	31.3	0.669
Subject 9	0.219	236	3.11	4.93	27.5	0.928
Subject 10	0.283	222	7.55	7.55	24.4	0.887
Subject 11	0.387	229	1.99	11.6	31.4	1.02
Subject 12	0.393	197	2.37	6.7	26.1	0.969
Subject 13	0.24	206	2.97	5.5	26.6	0.998
Subject 14	0.206	188	3.56	6.46	20.9	1.01
Subject 15	0.231	226	2.9	11	33.4	0.555
Subject 16	0.299	198	4.98	10.7	28.4	0.581
Subject 17	0.229	195	3.45	5.72	23.1	0.93
Subject 18	0.311	240	2.5	9.24	34.3	1.06
Subject 19	0.29	210	3.89	8.22	27.8	0.691
Subject 20	0.293	209	3.5	6.27	22.9	0.98
Subject 21	0.31	202	3.14	6.21	26.6	0.397
Subject 22	0.199	203	5.29	8.27	28.7	0.409
Subject 23	0.256	186	3.03	16.9	26.9	0.678
Subject 24	0.296	196	2.67	6.89	33.5	0.452
Subject 25	0.321	241	5.99	9.75	32.4	0.72
Subject 26	0.207	197	2.14	5.35	32.9	0.599
Subject 27	0.22	171	3.95	6.03	29.6	1.06
Subject 28	0.217	243	2.58	7.41	24.5	0.622
Subject 29	0.293	211	4.93	7.46	27.5	0.733
Subject 30	0.214	177	3.06	6.45	20.5	0.72
Subject 31	0.434	232	3.03	7.19	31.1	0.799
Subject 32	0.214	221	2.22	7.59	25.3	0.776
Subject 33	0.284	191	3.59	6.34	22.1	0.849
Subject 34	0.405	180	4.05	7.33	36.8	0.568
Subject 35	1.71	240	3.21	10.8	24.5	0.399
Subject 36	0.207	217	3.15	6.41	30.2	0.366
Subject 37	0.263	234	3.85	8.18	32.6	0.613
Subject 38	0.211	215	2.51	9.07	26.2	0.968
Subject 39	0.232	192	3.66	19.1	24.7	0.706
Subject 40	0.236	185	2.08	7.68	34.5	1.08
Subject 41	0.432	209	1.82	8.08	38.9	1.36
Subject 42	0.208	175	1.92	8.28	26.2	0.682
Subject 43	0.248	195	5.65	8.46	32.1	0.499
Subject 44	0.249	189	2.23	6.5	27.1	1.13
Subject 45	0.266	180	2.36	7.42	27.6	0.495

Subject 46	0.215	214	2	7.93	28.5	0.823
Subject 47	0.269	188	3.13	5.74	29	0.776
Subject 48	0.253	210	2.21	9.24	29.6	0.739
Subject 49	0.256	199	2.61	8.17	26.5	0.901
Subject 50	0.273	183	2.16	23.2	28.3	0.673
Subject 51	0.33	187	4.97	6.82	25.4	1.07
Subject 52	0.279	195	2.74	4.8	26.9	0.629
Subject 53	0.353	216	1.47	8.86	31	0.631
